# Integrating plasma cell‐free DNA with clinical laboratory results enhances the prediction of critically ill patients with COVID‐19 at hospital admission

**DOI:** 10.1002/ctm2.966

**Published:** 2022-07-15

**Authors:** Yong Bai, Fang Zheng, Tongda Zhang, Qiuhong Luo, Yuxue Luo, Ruilong Zhou, Yan Jin, Ying Shan, Jiehui Cheng, Zhimin Yang, Lingguo Li, Haiqiang Zhang, Yan Zhang, Jianhua Yin, Mingyan Fang, Dongsheng Chen, Fanjun Cheng, Xin Jin

**Affiliations:** ^1^ BGI‐Shenzhen Shenzhen Guangdong China; ^2^ Department of Pediatrics, Union Hospital, Tongji Medical College Huazhong University of Science and Technology Wuhan Hubei China; ^3^ College of Life Sciences University of Chinese Academy of Sciences Beijing China; ^4^ Department of Emergency Medicine, Union Hospital, Tongji Medical College Huazhong University of Science and Technology Wuhan Hubei China; ^5^ Guangdong Hospital of Traditional Chinese Medicine Zhuhai Guangdong China; ^6^ State Key Laboratory of Dampness Syndrome of Chinese Medicine The Second Affiliated Hospital of Guangzhou University of Chinese Medicine Guangzhou Guangdong China; ^7^ Department of Hematology, Union Hospital, Tongji Medical College Huazhong University of Science and Technology Wuhan Hubei China; ^8^ School of Medicine South China University of Technology Guangzhou Guangdong China

Dear Editor,

Owing to the substantial clinical heterogeneity of patients infected with SARS‐CoV‐2,^1^, ^2^ factors primarily relying upon clinical and/or laboratory parameters are yet inadequate to accurately predict COVID‐19 patients evolving to severe or critical illness at early stage.^3^, ^4^ Recent studies have revealed an elevated level of cell‐free DNA (cfDNA) in plasma in severe COVID‐19 patients due to massive cell death or irreversible multiorgan injuries during pathological conditions.^5^, ^6^ Therefore, the utilization of cfDNA profiling may benefit improving the COVID‐19 prediction and help understand molecular characteristics of the life‐threatening disease.^7^, ^8^ Herein, we developed an M2Model, a LightGBM‐based^9^ machine learning model with focal loss as an objective function to predict critical COVID‐19 at admission by jointly analysing multimodal data, including laboratory parameters and cfDNA profiles.

Laboratory results and blood samples were collected from a total of 399 consecutive hospitalized patients with COVID‐19 (345 noncritical and 54 critical patients; Table S1). Whole‐genome sequencing (WGS) was conducted on plasma cfDNA (Table S2), and we observed a slight shift towards shorter cfDNA fragments in critical patients compared to noncritical patients (Figure S1B). We derived three types of features from the WGS data, including fragment length ratio (denoted as FRAGL), transcription start site coverage score (denoted as TSS) and frequency of 4‐nucleotide motifs at 5′ fragment ends (denoted as MOTIF). Together with laboratory results (denoted as LAB; Table S3), we acquired four feature‐type‐specific datasets with totally 510 features after data preprocessing (Figures 1A and S1A–D).

**FIGURE 1:**
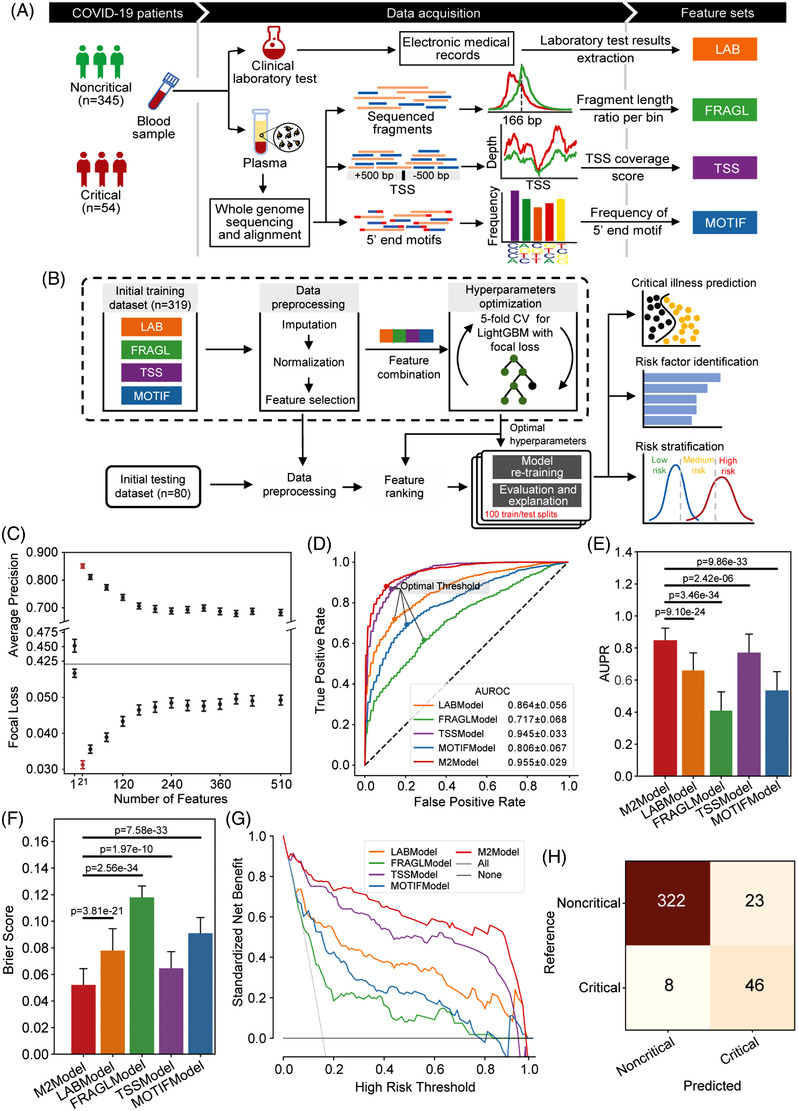
Feature generation and M2Model development for the prediction of critical COVID‐19. (A) Flow chart showing generation of four feature‐type specific datasets. Whole‐genome sequencing (WGS) was performed with an average depth of 13.6× on plasma cfDNA. The LAB features were extracted from the electronic medical records (EMRs), whereas features of FRAGL, TSS and MOTIF were derived from the WGS data. (B) Flow chart presenting the M2Model development to predict critical COVID‐19 by integrating the four types of features. (C) Evaluation of the M2Model by average precision score and focal loss with respect to the number of features that were ranked using the SHapley Additive exPlanations (SHAP) algorithm (see the Supporting Information). Error bars in red represented the optimal top 21 features. Error bars: mean ± standard error (SE). (D–G) Comparison of prediction performance of different models in terms of (D) ROC (receiver operating characteristic) curves, (E) AUPR (area under the precision‐recall curve) scores, (F) Brier scores and (G) decision curve analysis. In (D), the optimal cut‐off threshold for each model was determined using Youden's *J* statistic. In (G), the decision curve of each model was presented using the mean of prediction probabilities across 100 iterations of random training/testing splits. Error bars: mean ± standard deviation (SD). Statistical test: two‐side Mann–Whitney *U* test. (H) Confusion matrix showing the overall performance of the M2Model on discrimination between critical and noncritical patients at admission, which was accomplished by calculating the mean of predicted probabilities across 100 iterations against the optimal cut‐off threshold as shown in (C)

By integrating previous four datasets, the M2Model was trained and evaluated using 100 random training/testing splits based on the optimal hyperparameters and ranked features (Figure 1B, Table S6). For comparison, we applied the same protocol to each dataset, leading to four additional single‐type feature‐based models. The top‐predictive features were finally selected once the corresponding model yielded the highest average precision but the lowest focal loss (Figure 1C and S2A–D). Consequently, the M2Model outperformed other single‐type feature‐based models in discriminating critical from noncritical COVID‐19, achieving the highest AUROC (area under ROC curve) of .955 ± .029 (mean ± SD; Figure 1D) and AUPR (area under precision‐recall curve) of .827 ± .153 (*p *< .0001; Figure 1E). The Brier score for calibration assessment of the M2Model reached the lowest value of .052 ± .025, suggesting its optimal representation of the true critical COVID‐19 likelihood (*p *< .0001; Figure 1F). Decision curve analysis and confusion matrix also demonstrated the superior prediction ability of the M2Model over other models (Figures 1G,H and S3A–D), with sensitivity of 85.19% (95% confidence interval [CI], 63.6%–100.0%), specificity of 93.33% (95% CI, 86.2%–98.6%), PPV (positive predictive value) of 66.67% (95% CI, 48.8%–88.9%), NPV (negative predictive value) of 97.58% (95% CI, 94.0%–100.0%) and MCC (Matthews correlation coefficient) of 71.02% (95% CI, 49.8%–88.8%) (Table S4).

Although only 21 (4 LAB and 17 TSS) of 510 combined features (4.12%) were identified as top‐predictive features by the M2Model (Figure 2A), they accounted for 37.9% of total feature importance (Figure 2B). Remarkably, TSS features alone contributed the most towards critical COVID‐19 prediction (Figure 2C). Visualization of these 21 features showed complex non‐linear functions learned by the M2Model (Figures 2D and S4, S5). Additionally, we also analysed the top features identified by the single‐type feature‐based models (Figures S6–S9).

**FIGURE 2:**
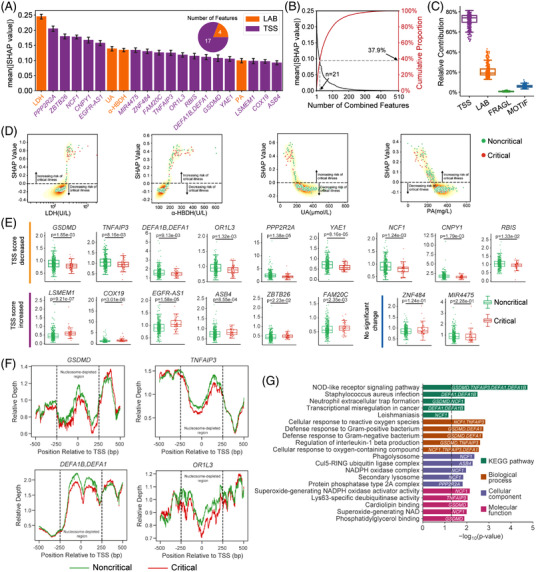
Top predictive features towards critical illness of COVID‐19 prediction identified by the M2Model. (A) Top 21 features prioritized by M2Model and ranked by the mean absolute SHapley Additive exPlanations (SHAP) values (see the Supporting Information). The TSS features were represented by the TSS‐associated gene symbols. Error bars: mean ± standard error (SE). (B) (Left, black) mean absolute SHAP values for the ranked individual features in the mixed feature‐type dataset and (right, red) cumulative proportion of mean absolute SHAP values with respect to the number of ranked features. (C) The relative contribution of each type of features within the mixed feature‐type dataset towards critically ill COVID‐19 prediction. A point in the boxplot represented an individual patient (*n* = 399). Boxplots: each box corresponded to an interval from the 25th to 75th percentile (interquartile range, IQR) and the median, whiskers = 1.5 ×  IQR. (D) Non‐linear relationships between the risk of critical illness and the identified LAB features. LDH, lactate dehydrogenase; PA, prealbumin; UA, uric acid; α‐HBDH, α‐hydroxybutyrate dehydrogenase. Each point in the plots represents a patient in the dataset (*n* = 399). (E) Boxplots showing differences in the identified TSS features (*n* = 17) between critical and noncritical patients. Statistical test: two‐side Mann–Whitney *U* test. Boxplots: each box corresponded to an interval from the 25th to 75th percentile IQR and the median, whiskers = 1.5 ×  IQR. (F) Nucleosome‐depleted regions (NDRs) between −250 and +250 bp around TSSs of *GSDMD*, *TNFAIP3*, *DEFA1B* and *DEFA1* at chr19:50968972, and *OR1L3* showing lower relative depth in critical patients than in noncritical patients. (G) KEGG pathway and functional GO term enrichment analyses for TSS‐associated genes. The top five enriched KEGG pathways were shown as well as the top five enriched GO terms for each of three categories. The identified TSS‐associated gene symbols were marked on the corresponding KEGG pathway or GO term. Black dashed line: *p *= .05

Of particular interest were the above 17 TSS features, of which 9 were significantly lower in critical than noncritical patients (*p *< .05; Figure 2E), reflecting a great loss of coverage depth in nucleosome‐depleted regions around these TSSs (Figures 2F and S10A). The low values of TSS features in critical patients could be linked to up‐regulated expression of the TSS‐associated genes, mainly resulting from the nucleosome occupancy for expressed genes^10^ (see the Supporting Information). Pathway and functional enrichment analyses of these genes showed significant correlations with immune‐related responses (*p *< .05; Figure 2G). For example, genes with lower values of TSS features in critical patients such as *GSDMD, TNFAIP3, DEFA1* and *DEFA1B* at chr19:50968972 were enriched in the top‐ranked pathway of ‘NOD‐like receptor’, where the related proteins were tightly interactive with each other (Figure S11). Gene set enrichment analysis indicated that many of these TSS‐associated genes were significantly related to COVID‐19 (Table S5).

We next clustered all patients into three risk strata according to the cut‐off values for critical COVID‐19 at 98% sensitivity and 98% specificity (Figure 3A). We illustrated that our M2Model was able to early predict COVID‐19 patients at a risk of deteriorating towards critical illness. For instance, PU8354 was a noncritical patient at admission but deteriorated during hospitalization. Our M2Model exhibited 47% and 26.1% contributions towards critical COVID‐19 prediction at admission by the 4 laboratory parameters and 17 TSS features, respectively, leading to an increasing risk of progressing towards critical illness from a prior probability of 1.2% to a posterior of 74.3% (Figure 3B). Overall survival analysis showed that the high‐risk critical COVID‐19 patients required a significantly longer length of hospital stay than other two risk groups (*p *< .005; Figure 3C). The univariate Cox proportional hazard analysis with recovery as the end‐point showed that the majority of the identified features were significantly correlated to decreasing the risk of critical COVID‐19 (*p *< .05; Figure 3D). The Spearman correlation analysis also displayed the strong associations between these features and the three risk strata (Figure 3E). Hierarchical clustering analysis demonstrated that these features were able to yield distinct separation among the three risk groups (Figure 3F).

**FIGURE 3:**
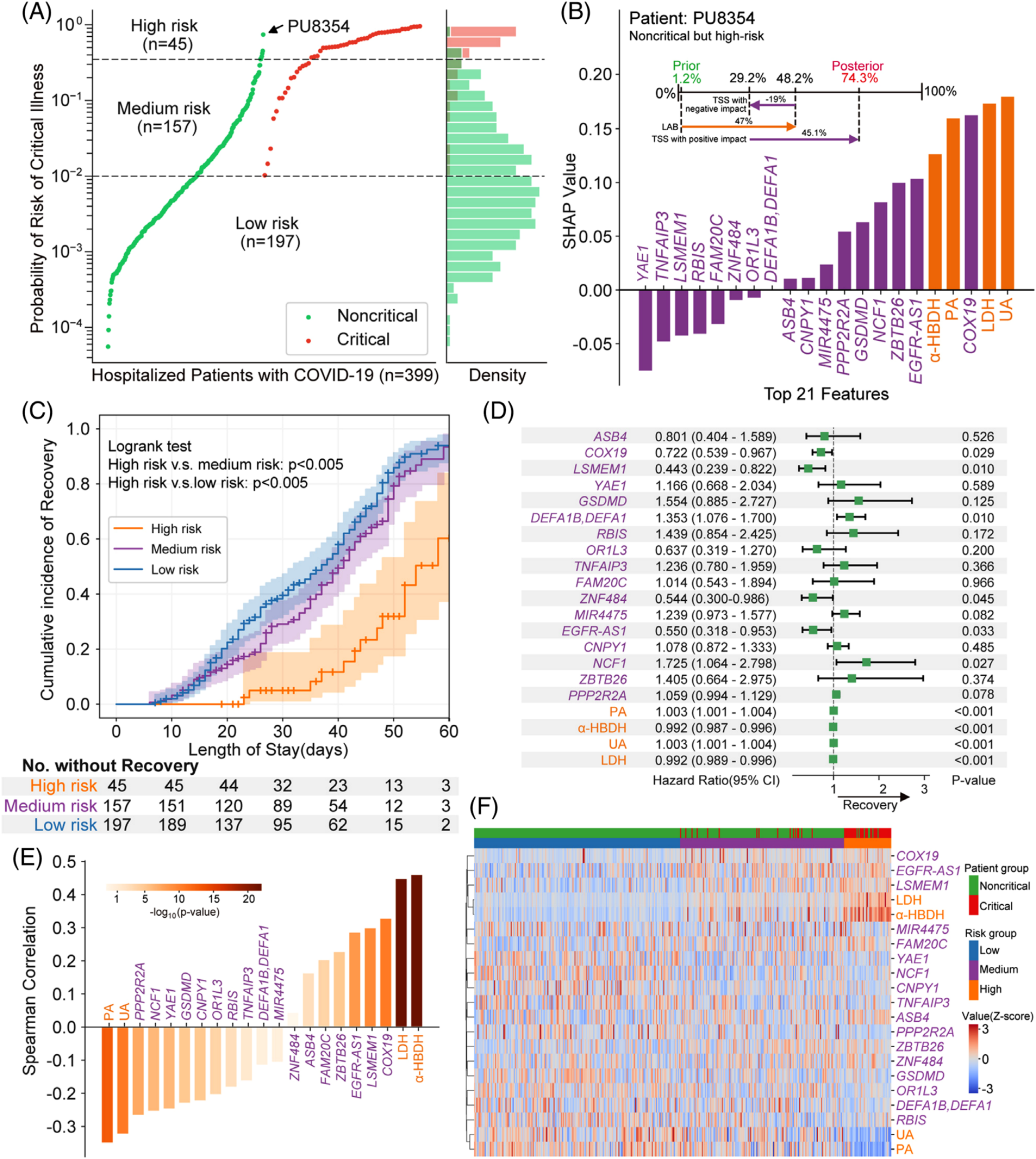
Risk stratification and prognostic utility of multimodal features identified by the M2Model. (A) Predicted probabilities for all patients determining three risk strata for critical illness of COVID‐19. A total of 197, 157 and 45 patients in the dataset were categorized into low‐, medium‐ and high‐risk strata, respectively, with true critical COVID‐19 patients of 0 (0%), 16 (10.19%) and 38 (84.44%), respectively. Patient PU8354 (male, 50‐year old) in the noncritical group, predicted to be high risk by M2Model, was highlighted. Upper dashed black line: cut‐off threshold for 98% sensitivity. Lower dashed black line: cut‐off threshold for 98% specificity. (B) Contribution of top 21 features towards critical COVID‐19 prediction for the patient PU8354. (Upper) The respective contribution of LAB and TSS features towards the evolution into the critical illness of COVID‐19 from a prior to a posterior probability. The prior probability was an expectation probability yielded by the M2Model across the training dataset. (Lower) SHapley Additive exPlanations (SHAP) values of the top 21 features for patient PU8354. (C) Kaplan–Meier curves for cumulative incidence of recovery in the three risk strata. The median duration to recovery was 29.0 days (95% CI, 8.9–56.2) in the low‐risk group, 35.0 days (95% CI, 9.9–59.0) in the medium‐risk group and 41.0 days (95% confidence interval [CI], 21.0–65.5) in the high‐risk group. The number of patients who had not yet recovered by time was shown. Shaded areas: 95% CI. Statistical test: log‐rank test. (D) The univariate Cox proportional hazard analysis using recovery as an end point. Hazard ratios (green squares) and 95% CI (horizontal lines) showed the prognostic utility of the 21 features (see the Supporting Information). (E) The Spearman rank correlation coefficients between the 21 features and the 3 risk strata. (F) Two‐way hierarchical clustering analysis of the top 21 features. All values were *z*‐scored

In summary, our M2Model was able to reach superior performance in predicting critical COVID‐19 at admission based on a compact subset of integrated laboratory parameters and TSS features. The TSS features, reflecting the open status of chromatin regions, displayed the most contribution to the prediction. The identified features with clinical and molecular characteristics had utilities for diagnostics and prognostics, and can serve as markers to monitor the effect of therapeutic interventions on critical COVID‐19. Additionally, our approach as a clinico‐genomic framework can be easily expanded towards early prediction of deteriorating patients who were initially infected with the emerging SARS‐CoV‐2 variants such as Omicron. We thereby anticipated that our M2Model had the potential to provide personalized management for individual patients with COVID‐19.

## FUNDING INFORMATION

The study was supported by the Guangdong‐Hong Kong Joint Laboratory on Immunological and Genetic Kidney Diseases (No. 2019B121205005) and the National Natural Science Foundation of China (Grant nos. 32171441 and 32000398).

## CONFLICT OF INTEREST

The authors declare that there is no conflict of interest that could be perceived as prejudicing the impartiality of the research reported.

## Supporting information

Supporting Information

Supporting tables information
