# Supplementary material for: Integrating plasma cell‐free DNA with clinical laboratory results enhances the prediction of critically ill patients with COVID‐19 at hospital admission
Source: Clin Transl Med. 2022 Jul 15;12(7):e966. doi: 10.1002/ctm2.966 (PMC9286531; doi:10.1002/ctm2.966)
Supplement: Supplementary file 1 — Supporting Information [file CTM2-12-0-s002.docx]

**SUPPORTING INFORMATION**

**Integrating plasma cell-free DNA with clinical laboratory results enhances prediction of critically ill patients with COVID-19 at hospital admission**

Yong Bai^1,*,†^, Fang Zheng^2, †^, Tongda Zhang^1,†^, Qiuhong Luo^1^, Yuxue Luo^1^, Ruilong Zhou^1,3^, Yan Jin^4^, Ying Shan^1^, JieHui Cheng^5^, Zhimin Yang^6^, Lingguo Li^1,3^, Haiqiang Zhang^1^, Yan Zhang^1^, Jianhua Yin^1^, Mingyan Fang^1^, Dongsheng Chen^1,*^, Fanjun Cheng^7,*^, Xin Jin^1,8,*^

^*^Correspondence email: baiyong@genomics.cn, jinxin@genomics.cn, chengfanjun001@sina.com, chendongsheng@genomics.cn

**Contents**

**Materials and Methods**

**Multimodal predictive features identified by M2Model**

**Univariate Cox proportional hazard analysis**

**Statistical analysis**

**Table S1**

**Table S2**

**Table S3**

**Table S4**

**Table S5**

**Table S6**

**Table S7**

(Table S7 was a Microsoft Excel spreadsheet file)

**Figure S1**

**Figure S2**

**Figure S3**

**Figure S4**

**Figure S5**

**Figure S6**

**Figure S7**

**Figure S8**

**Figure S9**

**Figure S10**

**Figure S11**

# Materials and Methods

**Patient Enrollment and Ethics**

This retrospective study was approved by the Institutional Review Board of BGI and the Medical Ethics Committee of Union Hospital, Tongji Medical College, Huazhong University of Science and Technology, Wuhan, China. All enrolled patients with written informed consent forms were laboratory-confirmed COVID-19 and admitted to the Union Hospital between January 15, 2020 and April 4, 2020. Patients were categorized into four groups (mild, moderate, severe and critical) according to the Diagnosis and Treatment Protocol for COVID-19 (Trial Version 7) issued by the General Office of the National Health Commission and the Office of the National Administration of Traditional Chinese Medicine. In this study, we combined mild, moderate and severe (not critical) groups and defined them as the noncritical group. We excluded patients with laboratory results missing, mismatched diagnoses, or lost to follow-up.

**Data collection**

The demographics, characteristics, and clinical laboratory results of patients in this study were extracted from electronic medical records (EMRs). Blood samples were collected from the remaining samples after clinical testing. For a patient with multiple clinical laboratory examinations during admission, we only collected blood sample and laboratory results available at the first clinical laboratory test (referred to as the initial laboratory test). We excluded the laboratory parameters with a missing ratio greater than 30% across all enrolled patients in the entire cohort.

**Sample processing and cfDNA extraction**

All blood samples collected in EDTA anticoagulation tubes were centrifuged at 1600g for 10 min at 4°C, the plasma layer was then transferred to an unused tube and stored at -80°C for later use. Each blood sample was processed into the required plasma within 6 hours after collection. Cell-free DNA was extracted from 200 µl plasma with MagPure Circulating DNA Kit (MD5432-02, Magen Biotechnology Co., Ltd., Guangzhou, China) on the automatic pipetting workstation of MGISP-96XL (MGI Tech Co., Ltd., Shenzhen, China) according to the manufacturer’s protocol. All the extraction was performed in a biosafety level 2 (BSL-2) laboratory, and technicians were following biosafety level 3 (BSL-3) protection standards.

**Sequencing library preparation**

The extracted cfDNA was used for further library construction followed by an MGIEasy Cell-free DNA Library Prep kit (MGI Tech Co., Ltd.) according to the manufacturer’s instructions. The concentration of the amplified cfDNA libraries was measured using Qubit dsDNA HS Assay Kit (Invitrogen, Cat. No. Q32854) with FLUOstar Omega microplate reader (BMG Labtech, Germany). The qualified high-quality sequencing libraries were conducted on the DNBSEQ platform (MGI Tech Co., Ltd.) for next-generation sequencing.

**Cell-free DNA sequencing and data processing**

All libraries were sequenced in the way of paired-end 100bp reads to generate raw data in FASTQ format approximately 51 Gb. For further clean data, we used SOAPnuke ^1^ (v1.5.0) software to remove adapters, filter low-quality reads and high ratio N base in the raw reads with default parameters. These preprocessed reads were subsequently mapped to the human reference genome (NCBI build GRCh38) using BWA ^2^ software with default parameters. PCR duplicates were removed using in-house programs after alignment, and then those filtered data were applied to cfDNA characteristics profiling. Consequently, the overall average sequencing depth of each sample was 13.6 $\times$ (**Table S2**).

**Features derived from cfDNA profiles**

To investigate the utility of cfDNA to predict critically ill patients with COVID-19, we derived three types of nongenetic features from cfDNA profiles. It should be notable that all features were calculated throughout the whole genome except chromosome Y. The three types of features were:

1. Fragment length ratio ^3^ (denote as FRAGL), defined as the proportion of sequenced fragment length ranging from 103bp to 259bp in every 10.4bp bin in the whole genome:

$\mathrm{FRGL}_{i}=\frac{\sum_{k=1}^{N} \delta(L_{i}\leq size of \mathrm{fragment}_{k}<L_{i+1})}{N}$ (1)

where $L_{i+1}=\left\lceil L_{i}+10.4 \right\rceil$, $L_{0}=103$, $i=0,1,...,15$, $\left\lceil\cdot\right\rceil$refers to rounding a number to the nearest integer, $\delta(x)=\left\{ \begin{aligned} 1, if x is true \\ 0, otherwise \end{aligned} \right.$, $N$ is the total number of fragments from an individual’s whole genome. It was noted that we filtered out fragments with length greater than 2000 bp.

1. Transcription start site coverage score ^4^ (denoted as TSS). We used sequencing reads in a 1000bp region centered on a TSS position (1k-TSS region) to calculate the TSS coverage score, which was defined as the read depth in the nucleosome-depleted region (NDR) between – 250 bp and +250 bp normalized by that in the 250 bp at each of end flanks:

$\mathrm{TSS}_{i}=\frac{\sum_{j=i-250}^{i+250} d_{j}}{(\sum_{j=i-500}^{i-250} d_{j}+\sum_{j=i+250}^{i+500} d_{j})}$ (2)

where $i$ is the TSS position. We acquired TSS positions by downloading http://hgdownload.cse.ucsc.edu/goldenpath/hg38/database/refGene.txt.gz. $d_{j}$ refers to the relative depth at genome position $j$,

$d_{j}=\frac{the number of reads at position j}{average depth of whole genome from an individual}$ (3)

In our analysis, we used TSS-associated gene symbols to represent the TSSs (**Table S7E**).

1. Frequency of 4-nucleotide motif at 5’ fragment ends ^5^ (denoted as MOTIF), referred to as the proportion of cfDNA fragments with the given motif in an individual’s whole genome:

$\mathrm{MOTIF}_{i}=\frac{\sum_{k=1}^{N} \delta( \mathrm{fragment}_{k} contains motif i)}{N}$ (4)

where $i\in\{AAAA, AAAT,...,GGGG\}$, $N$ is the total number of fragments from an individual’s WGS data. $\delta\left( \cdot\right)$ had the same definition as above.

Consequently, including clinical laboratory test results (denoted as LAB), there were a total of four feature-type specific datasets involved in the current study (**Table S7A-D**). LightGBM-based machine learning model with focal loss built upon a single-type dataset of LAB, FRAGL, TSS and MOTIF was referred to as LABModel, FRAGLModel, TSSModel and MOTIFModel, respectively.

**Data preprocessing**

**Data imputation.** Laboratory results had missing values even after exclusion and hence needed to be imputed before the downstream analysis. To accomplish this, we applied the missForest model ^6^ to impute missing values in a nonparametric fashion. The missForest iteratively used a random forest that was fitted on the observed part of data to predict the missing ones, without the need for validation due to the out-of-bag (OOB) imputation error estimation. The missForest model trained on the training set was further employed to impute the missing values of laboratory results in the independent test set.

**Data normalization.** Each feature was then normalized across the patients in the training set by removing the mean value and scaling to unit variance before model training. The estimated values of mean and standard deviation were then applied to the corresponding features in the independent test set.

**Feature selection.** Due to the high dimensionality of the TSS data in this study and hence may compromise the predictive capability of the model, we proposed a hybrid method to remove the redundant or irrelevant features. More specifically, the hybrid method combined the three different feature selection methods, including (i) mutual information (MI) based method, which was one of the widely used filter methods to select a subset of features; (ii) recursive feature elimination (RFE) method, which was a popular wrapper method to recursively remove features with the least weights assigned by an external estimator; (iii) Boruta ^7^ method, which was another widely used wrapper method to select a subset of features with importance greater than the highest feature importance that was calculated by permuting a copy of features across samples to destroy the relationship between features and class labels. A feature would be selected by the hybrid method if it was picked by all of the three methods simultaneously.

We used the MI-based method and the RFE method, both of which were implemented in the scikit-learn library (v 0.23.2), as well as the Boruta in the BorutaPy library (<https://github.com/scikit-learn-contrib/boruta_py> ). Moreover, random forests implemented in the scikit-learn library with default settings were employed to calculate feature importance for RFE and Boruta, respectively. The hybrid method was applied on the training set to obtain a subset of relevant features, whereby the corresponding subset of features from the independent testing set was also determined.

After data preprocessing, there were a total of 62 features in LAB dataset, 15 in FRAGL dataset, 177 in TSS dataset, and 256 in MOTIF dataset.

**Development of LightGBM-based machine learning model with focal loss**

We developed light gradient boosting machine (LightGBM ^8^), an advanced gradient boosting framework based on decision tree algorithm, to distinguish critically ill patients with COVID-19 from noncritical ones at admission. Due to the largely imbalanced dataset (i.e., the number of noncritically ill patients was much greater than critically ill ones) that may greatly compromise the predictive accuracy of the model, we further adopted focal loss^9^ as the objective function. Let $y_{i}\in\{0,1\}$ be true label of severity status of patient $i$ ($y_{i}=1$ if patient $i$ was critically ill), $z_{i}$ be initial output from LightGBM for patient $i$, and $p_{i} =\frac{1}{1+e^{-z_{i}}}$, the focal loss is then defined as:

$\mathcal{L}_{\mathrm{FL}}=-\frac{1}{N}\sum_{i=1}^{N} \left( y_{i}\times\alpha{(1-p_{i})}^{\gamma}\log p_{i}+(1-y_{i})\times(1-\alpha){p_{i}}^{\gamma}log (1-p_{i}) \right)$ (5)

where $\alpha$ and $\gamma$ are hyperparameters needed to be optimized, $\alpha\in[0,1]$, $\gamma\geq0$. $N$ is the total number of patients in our dataset.

To enable optimization of the focal loss on the course of LightGBM learning, we took the first-order derivative and the second-order derivative of $\mathcal{L}_{\mathrm{FL}}$ with respect to $z_{i}$, respectively, which yielded:

$\frac{\partial\mathcal{L}_{\mathrm{FL}}}{\partial z_{i}}=\left\{ \begin{aligned} \alpha\left( 1-p_{i} \right)^{\gamma}(\gamma p_{i}\log p_{i}+p_{i}-1), \mathrm{if} y_{i}=1 \\ \left( 1-\alpha\right){p_{i}}^{\gamma}\left[ \gamma(1-p_{i})\log\left( 1-p_{i} \right)-p_{i} \right] , \mathrm{otherwise} \end{aligned} \right.$ (6)

$\frac{\partial^{2}\mathcal{L}_{\mathrm{FL}}}{\partial{z_{i}}^{2}}=\left\{ \begin{aligned} \alpha\left( 1-p_{i} \right)^{\gamma}\left[ \frac{1-p_{i}-\gamma p_{i}\log p_{i}}{1-p_{i}}+\gamma\log p_{i}+\gamma+1 \right]\cdot p_{i}\left( 1-p_{i} \right),\mathrm{if} y_{i}=1 \\ \left( 1-\alpha\right){p_{i}}^{\gamma}\left[ \frac{-\gamma p_{i}+\gamma^{2}(1-p_{i})\log\left( 1-p_{i} \right)}{p_{i}}-1-\gamma\log\left( 1-p_{i} \right)-\gamma\right]\cdot p_{i}\left( 1-p_{i} \right), \mathrm{otherwise} \end{aligned} \right.$ (7)

**Data splitting and model training**

We first randomly split the entire dataset into an initial training set and testing set. The initial training set was used to optimize hyperparameters of a model in five-fold cross-validation. Once the optimal hyperparameters were determined, we trained the model with the optimal hyperparameters and features were then ranked in decreasing order of feature importance calculated using SHAP (SHapley Additive exPlanations) algorithm ^10^ based on the initial testing set. After that, we randomly split training/testing again with 100 iterations. At each iteration, we retrained models again with the optimal hyperparameters using the training set based on a sequence of the ranked features, and evaluated the model using the testing set. The final performance metrics of the model were averaged across the 100 iterations. We identified the final feature list and final model that reached the best performance in terms of the highest average precision but the lowest focal loss. We applied the randomly stratified sampling method to construct training and testing set in an 8:2 ratio of patients. As a result, the same proportions of critical and noncritical patients can be preserved in the training and testing sets.

**Hyperparameters optimization**

We applied Bayesian optimization to optimize hyperparameters for LightGBM with focal loss. Bayesian optimization is an approach that iteratively searches hyperparameter values to minimize cross-validation error based on past evaluation results. The cross-validation error (CV error) is approximated by a probabilistic surrogate function, whose next input point yielding the maximal value of a predefined acquisition function will be sampled from the hyperparameter space. In this study, we employed Hyperopt ^11^ (v 0.2.5) to perform Bayesian optimization with 200 iterations. The average value of negative area under the receiver operating characteristics (AUROCs) in stratified five-fold cross-validation from the training set was used as CV error to be minimized.

**Feature ranking using SHAP value**

We adopted the SHAP (SHapley Additive exPlanations) value ^10^ (v 0.39.0) to rank the importance of individual features towards predicting the criticality of hospitalized patients with COVID-19. SHAP has been a widely used method to explain machine learning prediction based on game theory. A SHAP value quantifies the marginal contribution of a feature that influences the prediction probability of critical illness. Positive SHAP values are indicative of increasing risk while negative ones are indicative of decreasing risk. The mean absolute SHAP value per feature across all patients in the dataset provides the global feature importance. Features were initially ranked by the mean absolute SHAP values that were calculated using the independent testing test. The optimal number of features was then determined by the maximal mean of average precision scores evaluated across 100 iterations of random training/testing splits.

**Calculation of feature contribution**

We assessed relative contribution of different feature-type specific datasets towards critical COVID-19 illness prediction for each patient. The relative contribution of a feature-type specific dataset was calculated as a proportion of the sum of absolute SHAP values of a patient for each feature within the dataset over the total absolute SHAP values of the patient across all feature-type specific datasets.

**Enrichment analyses and PPI network**

The functional GO (gene ontology) terms and KEGG (kyoto encyclopedia of genes and genomes) pathways enrichment analyses were performed through Enrichr ^12,13^. We also used the Enrichr gene set enrichment analysis tool to conduct gene set enrichment analysis^14^. Besides, the protein-protein interaction (PPI) network was implemented using the STRING (search tool for the retrieval of interacting genes/proteins) database ^15^.

**Evaluation metrics**

Based on the independent testing datasets in the 100 iterations of random training/testing splits, the performance of the final model was evaluated using the following metrics:

1. Focal loss: equation (5).
2. Area under the receiver operating characteristic curve (AUROC).
3. Area under the precision-recall curve (AUPR).
4. Average precision score (AP):

$AP=\sum_{i} \left( R_{i}-R_{i-1} \right)P_{i}$ (8)

where $P_{i}$ and $R_{i}$ are precision and recall at the $i$th threshold.

1. Brier score (BS):

$\mathrm{BS}=\frac{1}{n}\sum_{i=1}^{n} {(y_{i}-p_{i})}^{2}$ (9)

where $n$ is the total number of patients independent testing dataset, $y_{i}$ is the true label of severity status of patient $i$ ($y_{i}=1$ if patient $i$ was critically ill) as defined above, $p_{i}$ is the predicted probability by the model. The BS measures the goodness of the predicted probability of being critical COVID-19, ranging from 0 to 1. A lower BS value implies more reliability of the classifier ^16^.

1. Matthews correlation coefficient (MCC):

$MCC=\frac{TP\times TN-FP\times FN}{\sqrt{(TP+FP)(TP+FN)(TN+FP)(TN+FN)}}$ (10)

where $\mathrm{TP}$ represents true positives, $\mathrm{FP}$ represents false positives, $\mathrm{TN}$ represents true negatives, $\mathrm{FN}$ represents false negatives.

1. Sensitivity (or recall):

$Sensitivity = \frac{\mathrm{TP}}{TP+FN}$ (11)

1. Specificity:

$Specificity=\frac{\mathrm{TN}}{TN+FP}$ (12)

1. Positive predictive value (PPV, or precision):

$PPV=\frac{\mathrm{TP}}{TP+FP}$ (13)

1. Negative predictive value (NPV):

$NPV=\frac{\mathrm{TN}}{TN+FN}$ (14)

# Multimodal predictive features identified by M2Model

Our M2Model identified the top 21 predictive features including 4 LAB and 17 TSS features. For the LAB features, we observed that lactate dehydrogenase (LDH) and α-hydroxybutyrate dehydrogenase (α-HBDH) positively correlated with aggravation of COVID-19 disease, and both were significantly increased in the critical patients compared to noncritical ones (**Figure S5A-B**). On the contrary, a significant reduction in the concentration of both uric acid (UA) and prealbumin (PA) was exhibited in critical patients (**Figure S5C-D**). These findings were consistent with previous reports ^17-20^. Both LDH and its isoenzyme α-HBDH can be released into the cell culture to increase levels during necrosis or apoptosis that is tightly related to innate and adaptive immunity, while the concentrations of UA and PA could be decreased in presence of inflammation and immune response activation, kidney dysfunction, or malnutrition ^20-22^. Indeed, SARS-CoV-2 may elicit inflammasomes activation, leading to macrophage pyroptosis, massive inflammation and cytokine storm, which plausibly contribute to multiorgan injuries in patients with severe or critical COVID-19 ^23-25^. This may in part shed light on the elevated levels of LDH and α-HBDH but decreased concentrations of UA and PA in patients with critical COVID-19. However, the role of immunopathological mechanisms in COVID-19 pathogenesis remains elusive, and comprehensive understanding requires further investigation.

For the TSS features, they can reflect the open status of chromatin regions as a result of occupancy of nucleosome or other binding proteins in plasma, and expressed genes can be inferred ^26^. Thus, they can indicate different biological processes between critical and noncritical patients, and the dynamic changes in cfDNA have the potential to provide insights into monitoring the pathobiological processes of the COVID-19 disease via liquid biopsies. A low cfDNA coverage depth in the nucleosome-depleted region (NDR) in the TSS region suggested that the associated gene was highly expressed ^26^. As expected, for instance, the decreased TSS coverage score of *GSDMD* may indicate that *GSDMD*-mediated inflammasome activations were dysregulated in critically ill patients ^27^, in line with previous observations that the capsid protein of the SARS-CoV-2 could bind to Gasdermin D encoded by this gene and inhibits pyroptosis in infected human monocytes ^28,29^. The low TSS coverage score of *TNFAIP3* in critically ill patients might suggest the high *TNFAIP3* expression in the severe stage of SARS-CoV-2 infection ^30^, which would likely be related to the increased activation in cytokine-mediated immune and inflammatory responses ^31^. As suggested by another study, drugs targeting TNFAIP3 might be potent in controlling COVID-19 hyper inflammation in the early phase of the disease ^32^. The declined TSS coverage scores of *DEFA1B* and *DEFA1* at chr19:50968972 might suggest that these anti-microbial and cytotoxic peptides were predictors of critical illness of COVID-19^33^. The low TSS coverage score of *OR1L3* might indicate destruction and regeneration of the olfactory receptors, and would likely lead to distortion of smell or parosmia in critical COVID-19 patients^34^.

The KEGG pathway enrichment analysis showed that *GSDMD*, *TNFAIP3*, *DEFA1* and *DEFA1B* were significantly enriched in "NOD-like receptor signaling pathway ", which was involved in foreign pathogens recognition and downstream pathway activation to stimulate immune responses ^35^. However, persistent activation or pathological conditions can lead to pathological damage to tissues and may even result in organ failure, infectious shock, and death ^23^. Other enriched pathways such as “Staphylococcus aureus infection”, “Neutrophil extracellular trap formation” and “Leishmaniasis” were also innate immune defense-related pathways. The GO term enrichment analysis also indicated that these genes identified by our M2Model were primarily enriched in biological processes which were strongly correlated with defense responses. Moreover, gene set enrichment analysis showed that the identified genes such as *NCF1*, *LSMEM1*, *TNFAIP3*, *ZNF484*, *GSDMD* and *PPP2R2A* were significantly related to COVID-19 (**Table S5**). Besides, the PPI network showed that the related proteins in the “NOD-like receptor signaling pathway” were tightly interactive with each other (**Figure S11**). For example, GSDMD interacting with CASP1 and CASP4 would cleave GSDMD to cause cell pyroptosis ^36,37^. The phosphatase 2 regulatory subunit A family members interacting with PPP2R2A were involved in the regulation of protein phosphatase 2A(PP2A), which played an important role in regulating inflammation ^38^ and were targeted by viruses to manipulate the host’s antiviral responses ^39^. The lower value of the TSS feature of *PPP2R2A* in critically ill patients might suggest the dysregulation activity of regulatory T cells (Tregs) modulated by PP2A, leading to the uncontrollable release of inflammatory cytokines in critical patients ^40^. RIG-I-like receptor signaling pathway members (IKBKB, TRAF6, TRAF2, RIPK1, FADD, IKBKG, TNF) that interacted with TNFAIP3 were targeted by SARS-CoV-2 nucleocapsid protein to inhibit the induction of interferon response ^41^.

# Univariate Cox proportional hazard analysis

The univariate Cox proportional hazard analysis with recovery as end-point showed that increased levels of TSS scores of DEFA1B, DEFA1 [hazard ratio (HR) 1.353; 95% CI, 1.076-1.700; *P*=0.010] and NCF1 (HR 1.725; 95% CI, 1.064-2.798; *P*=0.027), and laboratory parameters of PA (HR 1.003; 95% CI, 1.001-1.004; *P*<0.001) and UA (HR 1.003; 95% CI,1.001-1.004; *P*<0.001) were in favor of decreasing the risk of critical illness and recovering from COVID-19 (**Figure 3D**). In contrast, TSS features of *COX19* (HR 0.772; 95% CI, 0.539-0.967; *P*=0.029), *LSMEM1* (HR 0.443; 95% CI, 0.239-0.822; *P*=0.010), *ZNF484* (HR 0.544; 95% CI, 0.300-0.986; *P*=0.045) and *EGFR-AS1* (HR 0.550; 95% CI, 0.318-0.953; *P*=0.033), and clinical parameters of α-HBDH (HR 0.992; 95% CI, 0.987-0.996; *P*<0.001) and LDH (HR 0.992; 95% CI, 0.989-0.996; *P*<0.001) were risk factors of critical COVID-19. These findings reflected the prognostic utility of the markers for critical illness of COVID-19 as identified by the M2Model.

# Statistical analysis

All values were presented as means ± standard deviation (SD) unless specified otherwise. The two-side Mann-Whitney U test was used for the analyses of continuous variables, while the Pearson's $\chi^{2}$ test was applied to the categorical variables for comparison. *P* values of <0.05 were considered statistically significant for all analyses.

# References

1. Chen Y, Chen Y, Shi C, et al. SOAPnuke: a MapReduce acceleration-supported software for integrated quality control and preprocessing of high-throughput sequencing data. *Gigascience*. Jan 1 2018;7(1):1-6. doi:10.1093/gigascience/gix120

2. Li H, Durbin R. Fast and accurate short read alignment with Burrows-Wheeler transform. *Bioinformatics*. Jul 15 2009;25(14):1754-60. doi:10.1093/bioinformatics/btp324

3. Cristiano S, Leal A, Phallen J, et al. Genome-wide cell-free DNA fragmentation in patients with cancer. *Nature*. Jun 2019;570(7761):385-389. doi:10.1038/s41586-019-1272-6

4. Chen X, Wu T, Li L, et al. Transcriptional Start Site Coverage Analysis in Plasma Cell-Free DNA Reveals Disease Severity and Tissue Specificity of COVID-19 Patients. *Front Genet*. 2021;12:663098. doi:10.3389/fgene.2021.663098

5. Jiang P, Sun K, Peng W, et al. Plasma DNA End-Motif Profiling as a Fragmentomic Marker in Cancer, Pregnancy, and Transplantation. *Cancer Discov*. May 2020;10(5):664-673. doi:10.1158/2159-8290.CD-19-0622

6. Stekhoven DJ, Buhlmann P. MissForest--non-parametric missing value imputation for mixed-type data. *Bioinformatics*. Jan 1 2012;28(1):112-8. doi:10.1093/bioinformatics/btr597

7. Kursa MB, Rudnicki WR. Feature Selection with theBorutaPackage. *Journal of Statistical Software*. 2010;36(11)doi:10.18637/jss.v036.i11

8. Ke G, Meng Q, Finley T, et al. Lightgbm: A highly efficient gradient boosting decision tree. *Advances in neural information processing systems*. 2017;30:3146-3154.

9. Lin T-Y, Goyal P, Girshick R, He K, Dollár P. Focal loss for dense object detection. 2017:2980-2988.

10. Lundberg SM, Erion G, Chen H, et al. From Local Explanations to Global Understanding with Explainable AI for Trees. *Nat Mach Intell*. Jan 2020;2(1):56-67. doi:10.1038/s42256-019-0138-9

11. Bergstra J, Yamins D, Cox D. Making a science of model search: Hyperparameter optimization in hundreds of dimensions for vision architectures. PMLR; 2013:115-123.

12. Chen EY, Tan CM, Kou Y, et al. Enrichr: interactive and collaborative HTML5 gene list enrichment analysis tool. *BMC Bioinformatics*. Apr 15 2013;14:128. doi:10.1186/1471-2105-14-128

13. Kuleshov MV, Jones MR, Rouillard AD, et al. Enrichr: a comprehensive gene set enrichment analysis web server 2016 update. *Nucleic Acids Res*. Jul 8 2016;44(W1):W90-7. doi:10.1093/nar/gkw377

14. Xie Z, Bailey A, Kuleshov MV, et al. Gene Set Knowledge Discovery with Enrichr. *Curr Protoc*. Mar 2021;1(3):e90. doi:10.1002/cpz1.90

15. Szklarczyk D, Gable AL, Lyon D, et al. STRING v11: protein-protein association networks with increased coverage, supporting functional discovery in genome-wide experimental datasets. *Nucleic Acids Res*. Jan 8 2019;47(D1):D607-D613. doi:10.1093/nar/gky1131

16. Rufibach K. Use of Brier score to assess binary predictions. *J Clin Epidemiol*. Aug 2010;63(8):938-9; author reply 939. doi:10.1016/j.jclinepi.2009.11.009

17. Zhao D, Yao F, Wang L, et al. A Comparative Study on the Clinical Features of Coronavirus 2019 (COVID-19) Pneumonia With Other Pneumonias. *Clin Infect Dis*. Jul 28 2020;71(15):756-761. doi:10.1093/cid/ciaa247

18. Jiang H, Cheng H, Cao Q, et al. Clinical features, laboratory findings and persistence of virus in 10 children with coronavirus disease 2019 (COVID-19). *Biomed J*. Mar 2021;44(1):94-100. doi:10.1016/j.bj.2020.10.007

19. Liu YM, Xie J, Chen MM, et al. Kidney Function Indicators Predict Adverse Outcomes of COVID-19. *Med (N Y)*. Jan 15 2021;2(1):38-48 e2. doi:10.1016/j.medj.2020.09.001

20. Zinellu A, Mangoni AA. Serum Prealbumin Concentrations, COVID-19 Severity, and Mortality: A Systematic Review and Meta-Analysis. *Frontiers in Medicine*. 2021;8doi:10.3389/fmed.2021.638529

21. Shi Y, Evans JE, Rock KL. Molecular identification of a danger signal that alerts the immune system to dying cells. *Nature*. 2003;425(6957):516-521. doi:10.1038/nature01991

22. Werion A, Belkhir L, Perrot M, et al. SARS-CoV-2 causes a specific dysfunction of the kidney proximal tubule. *Kidney Int*. Nov 2020;98(5):1296-1307. doi:10.1016/j.kint.2020.07.019

23. Yap JKY, Moriyama M, Iwasaki A. Inflammasomes and Pyroptosis as Therapeutic Targets for COVID-19. *The Journal of Immunology*. 2020;205(2):307-312. doi:10.4049/jimmunol.2000513

24. Fajgenbaum DC, June CH. Cytokine Storm. *N Engl J Med*. Dec 3 2020;383(23):2255-2273. doi:10.1056/NEJMra2026131

25. Consiglio CR, Cotugno N, Sardh F, et al. The Immunology of Multisystem Inflammatory Syndrome in Children with COVID-19. *Cell*. Nov 12 2020;183(4):968-981 e7. doi:10.1016/j.cell.2020.09.016

26. Ulz P, Thallinger GG, Auer M, et al. Inferring expressed genes by whole-genome sequencing of plasma DNA. *Nat Genet*. Oct 2016;48(10):1273-8. doi:10.1038/ng.3648

27. Junqueira C, Crespo A, Ranjbar S, et al. SARS-CoV-2 infects blood monocytes to activate NLRP3 and AIM2 inflammasomes, pyroptosis and cytokine release. *Res Sq*. Aug 11 2021;doi:10.21203/rs.3.rs-153628/v1

28. Ma J, Zhu F, Zhao M, et al. SARS-CoV-2 nucleocapsid suppresses host pyroptosis by blocking Gasdermin D cleavage. *EMBO J*. Sep 15 2021;40(18):e108249. doi:10.15252/embj.2021108249

29. Vora SM, Lieberman J, Wu H. Inflammasome activation at the crux of severe COVID-19. *Nat Rev Immunol*. Nov 2021;21(11):694-703. doi:10.1038/s41577-021-00588-x

30. Li Y, Duche A, Sayer MR, et al. SARS-CoV-2 early infection signature identified potential key infection mechanisms and drug targets. *BMC Genomics*. Feb 18 2021;22(1):125. doi:10.1186/s12864-021-07433-4

31. Vereecke L, Beyaert R, van Loo G. The ubiquitin-editing enzyme A20 (TNFAIP3) is a central regulator of immunopathology. *Trends Immunol*. Aug 2009;30(8):383-91. doi:10.1016/j.it.2009.05.007

32. Islam T, Rahman MR, Aydin B, Beklen H, Arga KY, Shahjaman M. Integrative transcriptomics analysis of lung epithelial cells and identification of repurposable drug candidates for COVID-19. *Eur J Pharmacol*. Nov 15 2020;887:173594. doi:10.1016/j.ejphar.2020.173594

33. Wilk AJ, Lee MJ, Wei B, et al. Multi-omic profiling reveals widespread dysregulation of innate immunity and hematopoiesis in COVID-19. *J Exp Med*. Aug 2 2021;218(8)doi:10.1084/jem.20210582

34. Saniasiaya J, Narayanan P. Parosmia post COVID-19: an unpleasant manifestation of long COVID syndrome. *Postgrad Med J*. Mar 31 2021;doi:10.1136/postgradmedj-2021-139855

35. Platnich JM, Muruve DA. NOD-like receptors and inflammasomes: A review of their canonical and non-canonical signaling pathways. *Arch Biochem Biophys*. Jul 30 2019;670:4-14. doi:10.1016/j.abb.2019.02.008

36. Ding J, Wang K, Liu W, et al. Pore-forming activity and structural autoinhibition of the gasdermin family. *Nature*. Jul 7 2016;535(7610):111-6. doi:10.1038/nature18590

37. Liu X, Zhang Z, Ruan J, et al. Inflammasome-activated gasdermin D causes pyroptosis by forming membrane pores. *Nature*. Jul 7 2016;535(7610):153-8. doi:10.1038/nature18629

38. Kozicky LK, Sly LM. Phosphatase regulation of macrophage activation. *Semin Immunol*. Aug 2015;27(4):276-85. doi:10.1016/j.smim.2015.07.001

39. Guergnon J, Godet AN, Galioot A, et al. PP2A targeting by viral proteins: a widespread biological strategy from DNA/RNA tumor viruses to HIV-1. *Biochim Biophys Acta*. Nov 2011;1812(11):1498-507. doi:10.1016/j.bbadis.2011.07.001

40. Sharabi A, Kasper IR, Tsokos GC. The serine/threonine protein phosphatase 2A controls autoimmunity. *Clin Immunol*. Jan 2018;186:38-42. doi:10.1016/j.clim.2017.07.012

41. Oh SJ, Shin OS. SARS-CoV-2 Nucleocapsid Protein Targets RIG-I-Like Receptor Pathways to Inhibit the Induction of Interferon Response. *Cells*. 2021;10(3)doi:10.3390/cells10030530

**Supplementary tables**

# Table S1

**Baseline characteristics of hospitalized patients with COVID-19 in noncritical group and critical group**. Of 399 hospitalized patients with COVID-19, 5 patients (1.25%) were diagnosed as mild COVID-19 at admission, 140 (35.09%) moderate, 200 (50.13%) severe and 54 (13.53%) critical. We further categorized the critically ill patients into the critical group while the rest of patients into the noncritical group. At the end-point of the study, 256 (64.16%) were discharged with recovery, 125 (31.33%) were still in-hospital and 18 (4.51%) were dead. LOS: length of hospital stay, SysBP: systolic blood pressure, DiaBP: diastolic blood pressure, DM: diabetes mellitus, CHD: coronary heart disease, COPD: chronic obstructive pulmonary disease, HBV: hepatitis B virus, CKD: chronic kidney disease. Continuous variables are presented as mean ± standard deviation (SD) and the two-side Mann–Whitney U test is used to conduct a comparison. Categorical variables are expressed as count (percentage) and the Pearson's $\chi^{2}$ test is applied to compare.

|  | Total  (n=399) | Noncritical  (n=345) | Critical  (n=54) | P-value |
| --- | --- | --- | --- | --- |
| Male | 197(49.37%) | 162(46.96%) | 35(64.81%) | 0.0218 |
| Age(years) | 61.17±13.87 | 60.27±14.02 | 66.93±11.37 | 0.0010 |
| LOS(days) | 32.83±14.42 | 31.76±14.38 | 39.67±12.84 | 0.0001 |
| Physiological signs at hospital admission | | | | |
| SysBP(mmHg)* | 133.21±18.95 | 132.98±19.16 | 134.69±17.68 | 0.0808 |
| DiaBP(mmHg)# | 81.30±12.04 | 81.13±11.81 | 82.41±13.52 | 0.1657 |
| Highest body temperature(°C)§ | 37.89±1.16 | 37.84±1.15 | 38.22±1.15 | 0.0036 |
| Respiration rate(breaths per minute)¶ | 22.15±4.61 | 21.99±4.40 | 23.20±5.77 | 0.0308 |
| Heart rate(beats per minute)† | 90.94±16.02 | 90.89±16.04 | 91.30±15.99 | 0.4030 |
| Symptoms at hospital admission | | | | |
| Cough | 270(67.67%) | 232(67.25%) | 38(70.37%) | 0.7642 |
| Fever‡ | 260(65.16%) | 221(61.16) | 39(72.22%) | 0.3090 |
| Fatigue | 168(42.11%) | 141(40.87%) | 27(50%) | 0.2647 |
| Chest tightness | 162(40.60%) | 133(38.55%) | 29(53.70%) | 0.0501 |
| Poor appetite | 127(31.83%) | 111(32.17%) | 16(29.63%) | 0.8289 |
| Sputum | 118(29.57%) | 101(29.28%) | 17(31.48%) | 0.8650 |
| Muscle ache | 70(17.54%) | 62(17.97%) | 8(14.81%) | 0.7079 |
| Diarrhea | 63(15.79%) | 51(14.78%) | 12(22.22%) | 0.2327 |
| Vomiting | 28(7.02%) | 27(7.83%) | 1(1.85%) | 0.1896 |
| Headache | 28(7.02%) | 19(5.51%) | 9(16.67%) | 0.0070 |
| Dizziness | 27(6.77%) | 21(6.09%) | 6(11.11%) | 0.2822 |
| Pharyngalgia | 17(4.26%) | 16(4.64%) | 1(1.85%) | 0.5618 |
| Palpitation | 15(3.76%) | 12(3.48%) | 3(5.56%) | 0.7177 |
| Dyspnea | 12(3.01%) | 9(2.61%) | 3(5.56%) | 0.4529 |
| Night sweating | 10(2.51%) | 9(2.61%) | 1(1.85%) | 0.8908 |
| Chest pain | 10(2.51%) | 7(2.03%) | 3(5.56%) | 0.2831 |
| Rhinorrhoea | 9(2.26%) | 9(2.61%) | 0(0%) | 0.4791 |
| Hemoptysis | 7(1.75%) | 4(1.16%) | 3(5.56%) | 0.0835 |
| Abdominal pain | 3(0.75%) | 3(0.87%) | 0(0%) | 0.8375 |
| Comorbidities | | | | |
| Hypertension | 152(38.10%) | 122(35.36%) | 30(55.56%) | 0.0071 |
| DM | 76(19.05%) | 66(19.13%) | 10(18.52%) | 0.9363 |
| CHD | 53(13.28%) | 43(12.46%) | 10 (18.52%) | 0.3157 |
| Tumor | 20(5.01%) | 17(4.93%) | 3(5.56%) | 0.8897 |
| Cerebral infarction | 16(4.01%) | 13(3.77%) | 3(5.56%) | 0.8029 |
| Chronic bronchitis | 12(3.01%) | 10(2.90%) | 2(3.70%) | 0.9153 |
| COPD | 11(2.76%) | 7(2.03%) | 4(7.41%) | 0.0722 |
| Asthma | 4(1.00%) | 4(1.16%) | 0(0%) | 0.9516 |
| HBV | 4(1.00%) | 4(1.16%) | 0(0%) | 0.9516 |
| Tuberculosis | 3(0.75%) | 2(0.58%) | 1(1.85%) | 0.8735 |
| CKD | 3(0.75%) | 1(0.29%) | 2(3.70%) | 0.0638 |
| Smoking | 17(4.26%) | 13(3.77%) | 4(7.41%) | 0.3849 |
| * Missing in 11 noncritical patients and 3 critical patients. # Missing in 12 noncritical patients and 3 critical patients. § Missing in 3 noncritical patients and 2 critical patients. ¶ Missing in 4 noncritical patients and 3 critical patients. † Missing in 3 noncritical patients and 1 critical patient. ‡ Determined when body temperature was greater than 37.3°C. | | | | |

# Table S2

**Statistics of whole genome sequencing (WGS) reads.** Plasma sample for each individual was obtained from the initial laboratory test during admission. A total of 399 plasma samples were collected. The overall average sequencing depth of each plasma sample was 13.6 $\times$. bp: base pairs.

| **PID** | **Raw reads** | **Raw bases (bp)** | **Mapped reads** | **Mapping rate** | **Uniquely mapped reads** | **Uniquely mapping rate** | **Duplicate reads** | **Duplicate rate** | **Average depth** |
| --- | --- | --- | --- | --- | --- | --- | --- | --- | --- |
| PU5635 | 358858869 | 35867513619 | 356436460 | 99.32% | 337937680 | 94.81% | 12581381 | 3.53% | 11.26 |
| PU1254 | 316280008 | 31559354390 | 315719118 | 99.82% | 295265118 | 93.52% | 81646377 | 25.86% | 7.61 |
| PU1263 | 474332749 | 47402969454 | 474052904 | 99.94% | 447698290 | 94.44% | 33436499 | 7.05% | 14.42 |
| PU1266 | 711727076 | 71128888378 | 711181526 | 99.92% | 676048801 | 95.06% | 161770486 | 22.75% | 17.96 |
| PU1267 | 623126827 | 62274472248 | 622674641 | 99.93% | 598005108 | 96.04% | 391160179 | 62.82% | 7.55 |
| PU1269 | 513257661 | 51289969861 | 512867134 | 99.92% | 492559816 | 96.04% | 171323387 | 33.41% | 11.16 |
| PU1278 | 526946841 | 52655473894 | 526550302 | 99.92% | 497736334 | 94.53% | 20750287 | 3.94% | 16.54 |
| PU1279 | 514443640 | 51410711422 | 513760060 | 99.87% | 484365403 | 94.28% | 94460685 | 18.39% | 13.71 |
| PU1281 | 476064727 | 47562239789 | 475892599 | 99.96% | 446257673 | 93.77% | 57308304 | 12.04% | 13.67 |
| PU1282 | 496264341 | 49591802606 | 481714476 | 97.07% | 454222720 | 94.29% | 125743339 | 26.10% | 11.63 |
| PU1285 | 610668675 | 61019128409 | 610179164 | 99.92% | 574917489 | 94.22% | 39395258 | 6.46% | 18.65 |
| PU1288 | 828970581 | 82819702038 | 828523894 | 99.95% | 777166723 | 93.80% | 22817051 | 2.75% | 26.32 |
| PU1289 | 486440226 | 48611632549 | 486149630 | 99.94% | 460441828 | 94.71% | 28954064 | 5.96% | 14.96 |
| PU1290 | 494451807 | 49396525766 | 494245690 | 99.96% | 475988072 | 96.31% | 46998405 | 9.51% | 14.60 |
| PU1294 | 746427318 | 74593478878 | 743621918 | 99.62% | 702204427 | 94.43% | 27696671 | 3.72% | 23.42 |
| PU1297 | 297182042 | 29691047726 | 297023187 | 99.95% | 276540387 | 93.10% | 36850944 | 12.41% | 8.50 |
| PU1299 | 789471638 | 78883424294 | 789030201 | 99.94% | 742209490 | 94.07% | 47743592 | 6.05% | 24.23 |
| PU1301 | 578768583 | 57838632607 | 578277176 | 99.92% | 544794894 | 94.21% | 30946706 | 5.35% | 17.91 |
| PU1303 | 569070391 | 56873309143 | 568332191 | 99.87% | 537154392 | 94.51% | 294213340 | 51.77% | 8.96 |
| PU1305 | 714927744 | 71447574659 | 714298932 | 99.91% | 670719485 | 93.90% | 396368983 | 55.49% | 10.39 |
| PU1309 | 349429726 | 34919667926 | 349232972 | 99.94% | 328912671 | 94.18% | 15137718 | 4.33% | 10.93 |
| PU1310 | 683897238 | 68344097831 | 683440714 | 99.93% | 645515005 | 94.45% | 49815026 | 7.29% | 20.73 |
| PU1311 | 475566087 | 47530033001 | 475147860 | 99.91% | 448787817 | 94.45% | 249109090 | 52.43% | 7.39 |
| PU1312 | 428277866 | 42801776572 | 427989208 | 99.93% | 403487058 | 94.28% | 14591995 | 3.41% | 13.53 |
| PU1313 | 390327786 | 39009134995 | 390017475 | 99.92% | 369168757 | 94.65% | 23859628 | 6.12% | 11.99 |
| PU1314 | 257542884 | 25739240423 | 257292790 | 99.90% | 243575616 | 94.67% | 18607091 | 7.23% | 7.82 |
| PU1315 | 391544008 | 39131326346 | 368845222 | 94.20% | 347863221 | 94.31% | 36052704 | 9.77% | 10.89 |
| PU1316 | 483929795 | 48363258936 | 483567209 | 99.93% | 458319485 | 94.78% | 38235140 | 7.91% | 14.57 |
| PU1317 | 493182397 | 49281651041 | 492886855 | 99.94% | 464458067 | 94.23% | 16186650 | 3.28% | 15.59 |
| PU1319 | 499872848 | 49950168102 | 499448702 | 99.92% | 468787975 | 93.86% | 25673593 | 5.14% | 15.49 |
| PU1320 | 382124295 | 38191968706 | 381863375 | 99.93% | 361717037 | 94.72% | 24562957 | 6.43% | 11.70 |
| PU1321 | 461992082 | 46168157287 | 461327914 | 99.86% | 434213827 | 94.12% | 40495116 | 8.78% | 13.77 |
| PU1324 | 719589886 | 71896240888 | 719018947 | 99.92% | 674503762 | 93.81% | 15721049 | 2.19% | 22.98 |
| PU1325 | 480974726 | 48065272815 | 472712554 | 98.28% | 447466494 | 94.66% | 26978277 | 5.71% | 14.58 |
| PU1326 | 336621359 | 33643959522 | 329411375 | 97.86% | 312509770 | 94.87% | 17142718 | 5.20% | 10.22 |
| PU1343 | 752865536 | 75239653433 | 752369305 | 99.93% | 711075270 | 94.51% | 27836488 | 3.70% | 23.70 |
| PU1344 | 596496878 | 59621510410 | 595924019 | 99.90% | 563049668 | 94.48% | 465045418 | 78.04% | 4.26 |
| PU1346 | 457241295 | 45694021394 | 456892348 | 99.92% | 431293317 | 94.40% | 23153962 | 5.07% | 14.19 |
| PU1348 | 112852632 | 11274673370 | 112814213 | 99.97% | 106375745 | 94.29% | 11618761 | 10.30% | 3.30 |
| PU1356 | 347541845 | 34731620265 | 337820272 | 97.20% | 317771388 | 94.07% | 19599828 | 5.80% | 10.41 |
| PU1409 | 378140108 | 37795037101 | 374085067 | 98.93% | 353616535 | 94.53% | 11867470 | 3.17% | 11.86 |
| PU1428 | 397883357 | 39765684201 | 390830011 | 98.23% | 370077873 | 94.69% | 32421427 | 8.30% | 11.73 |
| PU1430 | 575747553 | 57532943352 | 572703202 | 99.47% | 538808178 | 94.08% | 18114288 | 3.16% | 18.13 |
| PU1436 | 560603847 | 56027687108 | 558314224 | 99.59% | 524905349 | 94.02% | 19247718 | 3.45% | 17.64 |
| PU1452 | 873071975 | 87223898633 | 872082222 | 99.89% | 817167542 | 93.70% | 25012462 | 2.87% | 27.67 |
| PU1463 | 716954845 | 71619086493 | 715983657 | 99.86% | 667476468 | 93.23% | 37201967 | 5.20% | 22.13 |
| PU6586 | 621075411 | 62071972828 | 620745228 | 99.95% | 591993110 | 95.37% | 145367406 | 23.42% | 15.56 |
| PU6600 | 507029550 | 49166347040 | 506847903 | 99.96% | 466215539 | 91.98% | 14537110 | 2.87% | 14.69 |
| PU6624 | 274546918 | 27431284236 | 274263737 | 99.90% | 259297182 | 94.54% | 164579088 | 60.01% | 3.57 |
| PU6657 | 582098346 | 58164786607 | 581602806 | 99.91% | 547761104 | 94.18% | 50706327 | 8.72% | 17.35 |
| PU6663 | 582637016 | 58232724559 | 582343710 | 99.95% | 548616533 | 94.21% | 146063355 | 25.08% | 14.27 |
| PU6664 | 580903695 | 58054637309 | 580630798 | 99.95% | 548174902 | 94.41% | 24252688 | 4.18% | 18.20 |
| PU6669 | 386166498 | 38567555729 | 385815795 | 99.91% | 370051867 | 95.91% | 37890986 | 9.82% | 11.37 |
| PU6683 | 398518761 | 39829932242 | 390415348 | 97.97% | 367423815 | 94.11% | 33205883 | 8.51% | 11.69 |
| PU6685 | 365112047 | 36487220709 | 364814146 | 99.92% | 347829779 | 95.34% | 173853107 | 47.66% | 6.24 |
| PU6688 | 296333451 | 29615556250 | 296028013 | 99.90% | 282986642 | 95.59% | 16549496 | 5.59% | 9.15 |
| PU6690 | 499986264 | 49963904219 | 499588317 | 99.92% | 471700781 | 94.42% | 39218915 | 7.85% | 15.06 |
| PU6703 | 988226006 | 98758651860 | 987666248 | 99.94% | 938295204 | 95.00% | 139110587 | 14.08% | 27.74 |
| PU6711 | 1046686480 | 104467555979 | 1046118446 | 99.95% | 994907091 | 95.10% | 68341516 | 6.53% | 31.84 |
| PU6721 | 438144143 | 43788572490 | 437609759 | 99.88% | 412274547 | 94.21% | 29273234 | 6.69% | 13.37 |
| PU6731 | 388857816 | 38862526397 | 388495731 | 99.91% | 368102710 | 94.75% | 225647967 | 58.08% | 5.32 |
| PU6732 | 494641154 | 49440320981 | 494352676 | 99.94% | 472115599 | 95.50% | 214534957 | 43.40% | 9.15 |
| PU6733 | 1430282527 | 142564933607 | 1429656889 | 99.96% | 1353990956 | 94.71% | 84174638 | 5.89% | 43.62 |
| PU6740 | 843377630 | 84268521199 | 842771975 | 99.93% | 792986062 | 94.09% | 34287379 | 4.07% | 26.42 |
| PU6751 | 434131020 | 43379425877 | 433927216 | 99.95% | 410855436 | 94.68% | 32744013 | 7.55% | 13.12 |
| PU6754 | 709035352 | 70873955783 | 708513576 | 99.93% | 676313931 | 95.46% | 529682524 | 74.76% | 5.84 |
| PU6757 | 692628999 | 69182841816 | 692225737 | 99.94% | 659993736 | 95.34% | 77095192 | 11.14% | 20.08 |
| PU6759 | 220098250 | 21704971658 | 219998429 | 99.95% | 207238790 | 94.20% | 7491128 | 3.41% | 6.65 |
| PU6761 | 447445798 | 44708450789 | 447124026 | 99.93% | 428546096 | 95.85% | 28968318 | 6.48% | 13.68 |
| PU6762 | 504894415 | 50453407067 | 504416674 | 99.91% | 479456393 | 95.05% | 207724475 | 41.18% | 9.68 |
| PU6765 | 504263296 | 50392766816 | 496670158 | 98.49% | 469446641 | 94.52% | 37008183 | 7.45% | 15.03 |
| PU6767 | 390928373 | 39068414546 | 390616357 | 99.92% | 367990845 | 94.21% | 241793297 | 61.90% | 4.86 |
| PU6770 | 513519416 | 51323881081 | 510007324 | 99.32% | 482606961 | 94.63% | 14794698 | 2.90% | 16.21 |
| PU6778 | 499656306 | 49930022147 | 499363849 | 99.94% | 472220599 | 94.56% | 29435055 | 5.89% | 15.37 |
| PU6779 | 442477141 | 44216572626 | 442239989 | 99.95% | 417517580 | 94.41% | 24984701 | 5.65% | 13.65 |
| PU6783 | 1136502423 | 113585300878 | 1135897079 | 99.95% | 1084256393 | 95.45% | 283765761 | 24.98% | 27.86 |
| PU6784 | 973159519 | 97153975169 | 972368609 | 99.92% | 900275255 | 92.59% | 25314058 | 2.60% | 30.85 |
| PU6785 | 1005381316 | 100448531846 | 1004874215 | 99.95% | 941990427 | 93.74% | 326536460 | 32.50% | 22.14 |
| PU6786 | 458554074 | 45834303935 | 458193866 | 99.92% | 438801200 | 95.77% | 277312231 | 60.52% | 5.91 |
| PU6787 | 197922567 | 19775078116 | 197505235 | 99.79% | 185888036 | 94.12% | 95699722 | 48.45% | 3.32 |
| PU6788 | 427880951 | 42735733220 | 427619698 | 99.94% | 401536680 | 93.90% | 178655192 | 41.78% | 8.12 |
| PU6789 | 529519440 | 52922880020 | 528553144 | 99.82% | 503792463 | 95.32% | 425102145 | 80.43% | 3.37 |
| PU6790 | 266970174 | 26682425024 | 266390504 | 99.78% | 251012738 | 94.23% | 99556703 | 37.37% | 5.45 |
| PU6791 | 647937396 | 64759888500 | 647495700 | 99.93% | 612725987 | 94.63% | 450107530 | 69.52% | 6.44 |
| PU6794 | 623943828 | 62364989645 | 623482790 | 99.93% | 590347687 | 94.69% | 433472096 | 69.52% | 6.21 |
| PU6799 | 646704495 | 64626581511 | 646268875 | 99.93% | 610857527 | 94.52% | 27342358 | 4.23% | 20.25 |
| PU6803 | 642091780 | 64176025819 | 641623210 | 99.93% | 609600057 | 95.01% | 407437031 | 63.50% | 7.65 |
| PU6804 | 517439849 | 51712236065 | 517023433 | 99.92% | 489426228 | 94.66% | 266111559 | 51.47% | 8.20 |
| PU6807 | 550668457 | 55024450035 | 550067779 | 99.89% | 518108654 | 94.19% | 25303581 | 4.60% | 17.15 |
| PU6809 | 308955904 | 30874893435 | 308688743 | 99.91% | 290023876 | 93.95% | 16603807 | 5.38% | 9.56 |
| PU6812 | 509658818 | 50936947124 | 509212340 | 99.91% | 482235791 | 94.70% | 31106472 | 6.11% | 15.65 |
| PU6819 | 446689556 | 44642235031 | 446333713 | 99.92% | 421989120 | 94.55% | 33210778 | 7.44% | 13.52 |
| PU6820 | 658267168 | 65786693819 | 657836020 | 99.93% | 619316071 | 94.14% | 35254560 | 5.36% | 20.38 |
| PU6822 | 597955255 | 59752720103 | 597471028 | 99.92% | 568942751 | 95.23% | 152256796 | 25.48% | 14.54 |
| PU6824 | 536193690 | 53587596159 | 531162499 | 99.06% | 501502694 | 94.42% | 26680271 | 5.02% | 16.51 |
| PU6825 | 634727027 | 63446475837 | 634161057 | 99.91% | 603880151 | 95.23% | 420208307 | 66.26% | 6.99 |
| PU6829 | 611379610 | 61100272580 | 610968432 | 99.93% | 570886744 | 93.44% | 264031547 | 43.22% | 11.34 |
| PU6831 | 544017481 | 54346816854 | 543690281 | 99.94% | 516557982 | 95.01% | 24065836 | 4.43% | 16.97 |
| PU6836 | 461347309 | 46114500561 | 460907951 | 99.90% | 439273870 | 95.31% | 353638599 | 76.73% | 3.49 |
| PU6842 | 508039324 | 50778405826 | 507426169 | 99.88% | 480745495 | 94.74% | 253269225 | 49.91% | 8.31 |
| PU6844 | 1062626583 | 106178652896 | 1062152233 | 99.96% | 998986939 | 94.05% | 32377984 | 3.05% | 33.66 |
| PU6846 | 508222961 | 50723728771 | 507908123 | 99.94% | 473281268 | 93.18% | 67345200 | 13.26% | 14.36 |
| PU6849 | 479627150 | 47921718198 | 479201990 | 99.91% | 448450863 | 93.58% | 26248284 | 5.48% | 14.79 |
| PU6851 | 461719101 | 46142141878 | 461439340 | 99.94% | 434985535 | 94.27% | 26514378 | 5.75% | 14.23 |
| PU6853 | 477129167 | 47683291734 | 476563226 | 99.88% | 449384802 | 94.30% | 22419599 | 4.70% | 14.85 |
| PU6857 | 541441843 | 54107481856 | 541084170 | 99.93% | 510990898 | 94.44% | 33542265 | 6.20% | 16.60 |
| PU6864 | 659467423 | 65917530721 | 659014752 | 99.93% | 631621078 | 95.84% | 427361446 | 64.85% | 7.57 |
| PU6866 | 452193400 | 45192361784 | 451944884 | 99.95% | 427159051 | 94.52% | 24617345 | 5.45% | 13.98 |
| PU6867 | 570802033 | 57051337709 | 570395464 | 99.93% | 543343856 | 95.26% | 342317839 | 60.01% | 7.44 |
| PU6870 | 551449057 | 55117115357 | 551048572 | 99.93% | 521867783 | 94.70% | 333893745 | 60.59% | 7.10 |
| PU6871 | 520056199 | 51979747758 | 519729210 | 99.94% | 490557788 | 94.39% | 338968615 | 65.22% | 5.90 |
| PU6878 | 522970910 | 52258884098 | 522651734 | 99.94% | 490068978 | 93.77% | 13499869 | 2.58% | 16.65 |
| PU6881 | 400899710 | 40062570535 | 400639730 | 99.94% | 374780489 | 93.55% | 87163011 | 21.76% | 10.25 |
| PU6882 | 392492643 | 39226931800 | 392136988 | 99.91% | 368879792 | 94.07% | 11718063 | 2.99% | 12.45 |
| PU6884 | 433330862 | 43304981577 | 432868620 | 99.89% | 407723125 | 94.19% | 21752208 | 5.03% | 13.45 |
| PU6887 | 392750078 | 39249942089 | 392458835 | 99.93% | 371467821 | 94.65% | 25655719 | 6.54% | 12.00 |
| PU6894 | 327731087 | 32753503730 | 327390223 | 99.90% | 313596934 | 95.79% | 178544077 | 54.54% | 4.86 |
| PU6898 | 394379240 | 39408959322 | 393880743 | 99.87% | 370782886 | 94.14% | 25252618 | 6.41% | 12.05 |
| PU6900 | 505117427 | 50483350452 | 504670216 | 99.91% | 478955938 | 94.90% | 352202413 | 69.79% | 4.97 |
| PU6905 | 442793309 | 44250144950 | 442277700 | 99.88% | 417720646 | 94.45% | 55600080 | 12.57% | 12.65 |
| PU6907 | 617474072 | 61708571293 | 616580028 | 99.86% | 579165430 | 93.93% | 50754913 | 8.23% | 18.51 |
| PU6912 | 439653665 | 43936845470 | 423805043 | 96.40% | 400380992 | 94.47% | 37288254 | 8.80% | 12.64 |
| PU6913 | 501609323 | 50129515439 | 501318514 | 99.94% | 472359983 | 94.22% | 23273772 | 4.64% | 15.64 |
| PU6916 | 471793774 | 47121831834 | 470837833 | 99.80% | 445709719 | 94.66% | 45714397 | 9.71% | 13.84 |
| PU6924 | 557175005 | 55679730868 | 556572145 | 99.89% | 524332739 | 94.21% | 57744649 | 10.38% | 16.32 |
| PU6930 | 636205009 | 63594259663 | 635571954 | 99.90% | 602149251 | 94.74% | 482914724 | 75.98% | 4.98 |
| PU6931 | 335910572 | 33572173996 | 327002894 | 97.35% | 309794169 | 94.74% | 18257788 | 5.58% | 10.11 |
| PU6932 | 216513459 | 21635226381 | 216409048 | 99.95% | 203758730 | 94.15% | 36643902 | 16.93% | 5.88 |
| PU6933 | 369688939 | 36945007721 | 369539544 | 99.96% | 346097266 | 93.66% | 64307251 | 17.40% | 9.99 |
| PU6937 | 448344777 | 44804272108 | 447963714 | 99.92% | 422252259 | 94.26% | 27971488 | 6.24% | 13.74 |
| PU6946 | 439147473 | 43886909403 | 438825996 | 99.93% | 415244539 | 94.63% | 30534623 | 6.96% | 13.37 |
| PU6954 | 633738791 | 63337427705 | 630464778 | 99.48% | 593849987 | 94.19% | 23202127 | 3.68% | 19.87 |
| PU6958 | 561117268 | 56072266414 | 560679278 | 99.92% | 528117484 | 94.19% | 26737903 | 4.77% | 17.47 |
| PU6959 | 538700530 | 53829904288 | 538317885 | 99.93% | 504579705 | 93.73% | 200388818 | 37.22% | 11.04 |
| PU6960 | 283272352 | 28308093214 | 283047128 | 99.92% | 272744912 | 96.36% | 70734332 | 24.99% | 6.94 |
| PU6961 | 534226081 | 53387589319 | 533507929 | 99.87% | 502595254 | 94.21% | 55631876 | 10.43% | 15.63 |
| PU6963 | 530360787 | 53007373608 | 503627372 | 94.96% | 477044600 | 94.72% | 90120849 | 17.89% | 13.53 |
| PU6965 | 454926820 | 45461804525 | 454556501 | 99.92% | 427543354 | 94.06% | 154977510 | 34.09% | 9.80 |
| PU6966 | 581870326 | 58129470338 | 580969649 | 99.85% | 551673305 | 94.96% | 322076522 | 55.44% | 8.43 |
| PU6967 | 352370775 | 35087470511 | 352195338 | 99.95% | 330395641 | 93.81% | 53984142 | 15.33% | 9.61 |
| PU6968 | 358733154 | 35851740782 | 358428368 | 99.92% | 342161416 | 95.46% | 61105161 | 17.05% | 9.72 |
| PU6970 | 393677233 | 39344925403 | 393363410 | 99.92% | 371811180 | 94.52% | 16860315 | 4.29% | 12.33 |
| PU6977 | 415496055 | 41524904534 | 415250615 | 99.94% | 392839380 | 94.60% | 15736125 | 3.79% | 13.08 |
| PU6987 | 546885889 | 54664190617 | 546369335 | 99.91% | 521175075 | 95.39% | 388548404 | 71.11% | 5.15 |
| PU6988 | 484996445 | 48476419834 | 484613545 | 99.92% | 460991201 | 95.13% | 331651012 | 68.44% | 4.99 |
| PU6989 | 573192281 | 57282059364 | 572693678 | 99.91% | 539940711 | 94.28% | 19873585 | 3.47% | 18.10 |
| PU6993 | 620438668 | 62008292475 | 620075012 | 99.94% | 584230561 | 94.22% | 207447252 | 33.46% | 13.50 |
| PU7009 | 484694488 | 48436507981 | 484319534 | 99.92% | 456535666 | 94.26% | 11197819 | 2.31% | 15.48 |
| PU7010 | 563316504 | 56295004395 | 562935203 | 99.93% | 530731607 | 94.28% | 28314232 | 5.03% | 17.49 |
| PU7015 | 674172519 | 67363750165 | 673731569 | 99.93% | 634030007 | 94.11% | 21561837 | 3.20% | 21.32 |
| PU7018 | 374720981 | 37443682487 | 374566324 | 99.96% | 361700646 | 96.57% | 50453744 | 13.47% | 10.60 |
| PU7019 | 420331382 | 42005428075 | 420057584 | 99.93% | 397519884 | 94.63% | 22465787 | 5.35% | 13.00 |
| PU7029 | 511893621 | 51161416707 | 511513702 | 99.93% | 484204385 | 94.66% | 228091487 | 44.59% | 9.27 |
| PU7031 | 598715166 | 59831423297 | 598437561 | 99.95% | 563981249 | 94.24% | 74860925 | 12.51% | 17.14 |
| PU7036 | 673481735 | 67305263061 | 673116807 | 99.95% | 631354740 | 93.80% | 98338192 | 14.61% | 18.80 |
| PU7044 | 182276986 | 18106697836 | 182198732 | 99.96% | 173448798 | 95.20% | 6688011 | 3.67% | 5.61 |
| PU7059 | 701572620 | 70111702757 | 701224884 | 99.95% | 664871451 | 94.82% | 145531257 | 20.75% | 18.17 |
| PU7065 | 621528915 | 62124029862 | 621068719 | 99.93% | 588166379 | 94.70% | 336245064 | 54.14% | 9.32 |
| PU7071 | 465860065 | 46491775191 | 465627803 | 99.95% | 438184770 | 94.11% | 155853739 | 33.47% | 10.08 |
| PU7075 | 320896235 | 32071970067 | 320718154 | 99.94% | 302220750 | 94.23% | 8361657 | 2.61% | 10.22 |
| PU7077 | 238371101 | 23815622178 | 237965710 | 99.83% | 226737719 | 95.28% | 17682056 | 7.43% | 7.18 |
| PU7079 | 567149182 | 56681570466 | 565233068 | 99.66% | 531125163 | 93.97% | 334784813 | 59.23% | 7.53 |
| PU7083 | 754262138 | 75372725061 | 753796778 | 99.94% | 707540080 | 93.86% | 20721740 | 2.75% | 23.97 |
| PU7093 | 553968558 | 55319240137 | 553496702 | 99.91% | 526856474 | 95.19% | 56163876 | 10.15% | 16.21 |
| PU7097 | 560787534 | 56044323506 | 560466642 | 99.94% | 524656421 | 93.61% | 217880222 | 38.87% | 11.20 |
| PU7100 | 477045433 | 47669098923 | 476814855 | 99.95% | 450170076 | 94.41% | 35906425 | 7.53% | 14.42 |
| PU7103 | 413729343 | 40405761523 | 413552902 | 99.96% | 382397193 | 92.47% | 15250431 | 3.69% | 12.07 |
| PU7108 | 559238575 | 55889196747 | 558849762 | 99.93% | 527508259 | 94.39% | 38452759 | 6.88% | 17.03 |
| PU7111 | 459664265 | 45947252940 | 459448355 | 99.95% | 434722909 | 94.62% | 153251364 | 33.36% | 10.02 |
| PU7113 | 409415898 | 40916129418 | 409079830 | 99.92% | 384836241 | 94.07% | 12944655 | 3.16% | 12.97 |
| PU7134 | 488324913 | 48797271269 | 487931694 | 99.92% | 458918370 | 94.05% | 69344430 | 14.21% | 13.69 |
| PU7135 | 774835282 | 77389845019 | 774524217 | 99.96% | 706281876 | 91.19% | 184175725 | 23.78% | 19.25 |
| PU7145 | 269224823 | 26905458928 | 268894857 | 99.88% | 252892995 | 94.05% | 18116382 | 6.74% | 8.21 |
| PU7150 | 298827498 | 29696136135 | 298715629 | 99.96% | 283965645 | 95.06% | 7255252 | 2.43% | 9.35 |
| PU7153 | 751566923 | 75067074330 | 751225014 | 99.95% | 703538987 | 93.65% | 15710873 | 2.09% | 24.01 |
| PU7157 | 785071992 | 78459226169 | 784615863 | 99.94% | 745486053 | 95.01% | 77850503 | 9.92% | 23.12 |
| PU7164 | 525008535 | 52390954481 | 524280307 | 99.86% | 489432871 | 93.35% | 80233377 | 15.30% | 14.40 |
| PU7165 | 290080718 | 28928610378 | 289945845 | 99.95% | 277125733 | 95.58% | 44499518 | 15.35% | 7.90 |
| PU7166 | 424482246 | 42392866993 | 424172207 | 99.93% | 400701847 | 94.47% | 124582163 | 29.37% | 9.77 |
| PU7172 | 650216142 | 64968488349 | 649934409 | 99.96% | 610010935 | 93.86% | 11805507 | 1.82% | 20.86 |
| PU7176 | 369956327 | 36973007296 | 369569537 | 99.90% | 345408678 | 93.46% | 206841924 | 55.97% | 5.31 |
| PU7177 | 670091076 | 66966306015 | 669645146 | 99.93% | 631400884 | 94.29% | 37168249 | 5.55% | 20.70 |
| PU7194 | 501286093 | 49993877520 | 501008225 | 99.94% | 476301106 | 95.07% | 13866361 | 2.77% | 15.83 |
| PU7199 | 368463218 | 36747032557 | 368252052 | 99.94% | 350463831 | 95.17% | 81271423 | 22.07% | 9.28 |
| PU7200 | 666645993 | 66502130444 | 666267141 | 99.94% | 631132914 | 94.73% | 96324390 | 14.46% | 18.48 |
| PU7203 | 351149722 | 35097206334 | 350775139 | 99.89% | 329582106 | 93.96% | 13675640 | 3.90% | 11.03 |
| PU7206 | 660298979 | 66000069484 | 659866530 | 99.93% | 634773179 | 96.20% | 281664378 | 42.69% | 12.37 |
| PU7222 | 496070388 | 48299537817 | 495835767 | 99.95% | 455565723 | 91.88% | 29699387 | 5.99% | 14.03 |
| PU7223 | 621409186 | 62112459438 | 621039444 | 99.94% | 590652716 | 95.11% | 159662738 | 25.71% | 15.11 |
| PU7224 | 470009662 | 46968926960 | 469574549 | 99.91% | 442702591 | 94.28% | 61520748 | 13.10% | 13.33 |
| PU7229 | 565802199 | 56550611204 | 565357322 | 99.92% | 537336959 | 95.04% | 178295997 | 31.54% | 12.66 |
| PU7231 | 660720161 | 66027897719 | 660127443 | 99.91% | 622596030 | 94.31% | 39689360 | 6.01% | 20.30 |
| PU7234 | 453269066 | 45301481099 | 452935861 | 99.93% | 427598092 | 94.41% | 20326429 | 4.49% | 14.17 |
| PU7239 | 508378357 | 50800358363 | 508049867 | 99.94% | 479519547 | 94.38% | 23731881 | 4.67% | 15.84 |
| PU7248 | 379715102 | 37947989221 | 379411972 | 99.92% | 359363229 | 94.72% | 26991629 | 7.11% | 11.54 |
| PU7249 | 193694198 | 19233278948 | 193609624 | 99.96% | 184812142 | 95.46% | 6461789 | 3.34% | 5.99 |
| PU7250 | 485760229 | 48517060758 | 485480726 | 99.94% | 460591774 | 94.87% | 215000281 | 44.29% | 8.82 |
| PU7251 | 323195141 | 32303826361 | 322875565 | 99.90% | 308289206 | 95.48% | 217664267 | 67.41% | 3.44 |
| PU7257 | 383152340 | 38297601608 | 382810056 | 99.91% | 363036981 | 94.83% | 273631518 | 71.48% | 3.56 |
| PU7258 | 513345949 | 51309878148 | 512779651 | 99.89% | 491228745 | 95.80% | 378038554 | 73.72% | 4.39 |
| PU7259 | 482142678 | 48180863195 | 481538420 | 99.87% | 454184686 | 94.32% | 46392325 | 9.63% | 14.23 |
| PU7271 | 864494946 | 86205138819 | 863918582 | 99.93% | 809579195 | 93.71% | 110181035 | 12.75% | 24.40 |
| PU7279 | 311750209 | 31154587282 | 311544241 | 99.93% | 293972118 | 94.36% | 20377896 | 6.54% | 9.53 |
| PU7295 | 552617470 | 55227832729 | 546570537 | 98.91% | 513647010 | 93.98% | 25686420 | 4.70% | 17.03 |
| PU7297 | 395679863 | 39545880264 | 388011265 | 98.06% | 365796845 | 94.27% | 20984525 | 5.41% | 12.01 |
| PU7302 | 366955345 | 36672379664 | 366656805 | 99.92% | 344133871 | 93.86% | 155795468 | 42.49% | 6.89 |
| PU7303 | 266124654 | 26595990343 | 265935808 | 99.93% | 252316427 | 94.88% | 14694107 | 5.53% | 8.23 |
| PU7304 | 303496052 | 30330268666 | 303242845 | 99.92% | 286691441 | 94.54% | 11911785 | 3.93% | 9.53 |
| PU7310 | 552086775 | 55168970699 | 551721296 | 99.93% | 521215561 | 94.47% | 23998950 | 4.35% | 17.26 |
| PU7312 | 628063274 | 62761601110 | 627606203 | 99.93% | 591116316 | 94.19% | 404823043 | 64.50% | 7.26 |
| PU7313 | 773831428 | 77315539631 | 773378102 | 99.94% | 729415164 | 94.32% | 40952285 | 5.30% | 23.93 |
| PU7321 | 402164161 | 40189238430 | 401867849 | 99.93% | 380366282 | 94.65% | 31389061 | 7.81% | 12.12 |
| PU7323 | 485380498 | 48507102549 | 485187439 | 99.96% | 456316998 | 94.05% | 48450266 | 9.99% | 14.29 |
| PU7326 | 568070634 | 56763204999 | 567598466 | 99.92% | 535679084 | 94.38% | 32573809 | 5.74% | 17.49 |
| PU7333 | 605701650 | 60528997055 | 605319740 | 99.94% | 568326588 | 93.89% | 14163503 | 2.34% | 19.33 |
| PU7335 | 654238980 | 65395458257 | 653689535 | 99.92% | 623997818 | 95.46% | 363692882 | 55.64% | 9.48 |
| PU7337 | 606212826 | 60006592591 | 605940255 | 99.96% | 570007443 | 94.07% | 95877975 | 15.82% | 16.11 |
| PU7338 | 533615538 | 53329373987 | 533290039 | 99.94% | 504836315 | 94.66% | 19995842 | 3.75% | 16.80 |
| PU7339 | 440760258 | 44051417531 | 440512031 | 99.94% | 416393114 | 94.52% | 16704589 | 3.79% | 13.88 |
| PU7343 | 464916992 | 46467154021 | 464652501 | 99.94% | 438444787 | 94.36% | 117513522 | 25.29% | 11.37 |
| PU7344 | 370010683 | 35978059839 | 369809513 | 99.95% | 334940449 | 90.57% | 9612488 | 2.60% | 10.80 |
| PU7345 | 388183344 | 38799138223 | 387971790 | 99.95% | 372014384 | 95.89% | 157763997 | 40.66% | 7.53 |
| PU7349 | 390871476 | 39066247329 | 390419847 | 99.88% | 368252355 | 94.32% | 159354924 | 40.82% | 7.56 |
| PU7353 | 475181954 | 47492175032 | 474890877 | 99.94% | 450908589 | 94.95% | 240129533 | 50.57% | 7.67 |
| PU7354 | 484831020 | 48457861054 | 484365801 | 99.90% | 459206789 | 94.81% | 315707920 | 65.18% | 5.51 |
| PU7355 | 458849538 | 45861067007 | 458297341 | 99.88% | 432783774 | 94.43% | 74948758 | 16.35% | 12.55 |
| PU7358 | 569032098 | 56862668304 | 568699105 | 99.94% | 537774606 | 94.56% | 43822783 | 7.71% | 17.17 |
| PU7360 | 834930253 | 83424905669 | 834540155 | 99.95% | 786922886 | 94.29% | 28244409 | 3.38% | 26.36 |
| PU7363 | 494490980 | 49420394300 | 471256616 | 95.30% | 446065782 | 94.65% | 100416789 | 21.31% | 12.14 |
| PU7365 | 444737352 | 44442970760 | 444409530 | 99.93% | 421802872 | 94.91% | 155220542 | 34.93% | 9.45 |
| PU7368 | 327944287 | 32745763785 | 327779355 | 99.95% | 312770038 | 95.42% | 48480547 | 14.79% | 9.08 |
| PU7369 | 462420969 | 46213617379 | 460783096 | 99.65% | 436476476 | 94.72% | 12361075 | 2.68% | 14.68 |
| PU7371 | 256324876 | 24981247598 | 256190801 | 99.95% | 237643011 | 92.76% | 6959555 | 2.72% | 7.53 |
| PU7378 | 261537101 | 26127529964 | 261050140 | 99.81% | 243614076 | 93.32% | 59777870 | 22.90% | 6.56 |
| PU7379 | 462735029 | 46240794997 | 462327778 | 99.91% | 436015596 | 94.31% | 32466611 | 7.02% | 14.06 |
| PU7382 | 539429237 | 53908288322 | 539157063 | 99.95% | 509516219 | 94.50% | 30383990 | 5.64% | 16.65 |
| PU7383 | 300069051 | 29984965957 | 299704433 | 99.88% | 282208277 | 94.16% | 13203868 | 4.41% | 9.37 |
| PU7384 | 690615050 | 69028311199 | 690025025 | 99.91% | 647172457 | 93.79% | 368259143 | 53.37% | 10.52 |
| PU7391 | 485577179 | 48523530737 | 485164074 | 99.91% | 459460520 | 94.70% | 37024529 | 7.63% | 14.66 |
| PU7392 | 424824471 | 42456287311 | 424522827 | 99.93% | 408565010 | 96.24% | 98333275 | 23.16% | 10.66 |
| PU7393 | 571146663 | 57080136328 | 570733289 | 99.93% | 548671695 | 96.13% | 175422334 | 30.74% | 12.93 |
| PU7395 | 526489570 | 52615635375 | 526076712 | 99.92% | 498184358 | 94.70% | 35370065 | 6.72% | 16.06 |
| PU7398 | 746733835 | 74632410593 | 746247047 | 99.93% | 707298173 | 94.78% | 235315467 | 31.53% | 16.70 |
| PU7401 | 420913765 | 42063765330 | 420427587 | 99.88% | 396822477 | 94.39% | 41904204 | 9.97% | 12.39 |
| PU7402 | 474304121 | 47403273203 | 473946837 | 99.92% | 447865685 | 94.50% | 30254001 | 6.38% | 14.52 |
| PU7409 | 546707014 | 54636404227 | 546415842 | 99.95% | 517145943 | 94.64% | 23380716 | 4.28% | 17.12 |
| PU7411 | 369113486 | 36886744261 | 368770479 | 99.91% | 349534733 | 94.78% | 38027812 | 10.31% | 10.82 |
| PU7414 | 753382230 | 75291703816 | 745840265 | 99.00% | 707578956 | 94.87% | 37671141 | 5.05% | 23.18 |
| PU7423 | 376128524 | 37594517645 | 357800221 | 95.13% | 337830974 | 94.42% | 72317785 | 20.21% | 9.35 |
| PU7427 | 418522884 | 41826974956 | 417943658 | 99.86% | 392779110 | 93.98% | 49832059 | 11.92% | 12.04 |
| PU7428 | 584183834 | 58372481209 | 583788855 | 99.93% | 547263968 | 93.74% | 25864172 | 4.43% | 18.24 |
| PU7430 | 804193061 | 80357057978 | 800650384 | 99.56% | 756211853 | 94.45% | 33731450 | 4.21% | 25.07 |
| PU7431 | 491675266 | 49133538273 | 491279429 | 99.92% | 463472840 | 94.34% | 35628502 | 7.25% | 14.91 |
| PU7433 | 328803378 | 32860696980 | 328645286 | 99.95% | 310050254 | 94.34% | 84397080 | 25.68% | 7.99 |
| PU7434 | 446725918 | 44646213019 | 446311411 | 99.91% | 420233966 | 94.16% | 19932288 | 4.47% | 13.96 |
| PU7435 | 484358899 | 48402182051 | 483945496 | 99.91% | 455361007 | 94.09% | 24992666 | 5.16% | 15.01 |
| PU7436 | 825054156 | 82436646693 | 820960850 | 99.50% | 770137478 | 93.81% | 38671530 | 4.71% | 25.56 |
| PU7438 | 504237014 | 50386121923 | 503621325 | 99.88% | 473690848 | 94.06% | 68535012 | 13.61% | 14.22 |
| PU7442 | 603096316 | 60264023347 | 599743418 | 99.44% | 566623397 | 94.48% | 18919243 | 3.15% | 18.99 |
| PU7445 | 410603985 | 41040319065 | 403194594 | 98.20% | 383339322 | 95.08% | 32212445 | 7.99% | 12.15 |
| PU7446 | 386381993 | 38617517904 | 374892804 | 97.03% | 354697864 | 94.61% | 29540994 | 7.88% | 11.31 |
| PU7447 | 567722906 | 56735858755 | 543099988 | 95.66% | 514192501 | 94.68% | 92268815 | 16.99% | 14.75 |
| PU7448 | 348511726 | 34834395179 | 314488660 | 90.24% | 298253183 | 94.84% | 70730718 | 22.49% | 7.98 |
| PU8097 | 45863791 | 4581431061 | 45752581 | 99.76% | 43018825 | 94.02% | 7889829 | 17.24% | 1.24 |
| PU8107 | 592673524 | 59227770831 | 592217063 | 99.92% | 558084083 | 94.24% | 36916560 | 6.23% | 18.16 |
| PU8125 | 497426856 | 49713491120 | 497097325 | 99.93% | 471541889 | 94.86% | 25294174 | 5.09% | 15.44 |
| PU8128 | 521824340 | 52149798380 | 517470603 | 99.17% | 490086971 | 94.71% | 32404868 | 6.26% | 15.88 |
| PU8129 | 248441930 | 24829709674 | 247970260 | 99.81% | 233813625 | 94.29% | 66680234 | 26.89% | 5.92 |
| PU8130 | 137335449 | 13722857918 | 137272178 | 99.95% | 129594117 | 94.41% | 37063822 | 27.00% | 3.27 |
| PU8145 | 475834037 | 47558750398 | 475437048 | 99.92% | 449312785 | 94.51% | 358603619 | 75.43% | 3.81 |
| PU8146 | 524167204 | 52380526646 | 523941082 | 99.96% | 501423594 | 95.70% | 55355729 | 10.57% | 15.33 |
| PU8151 | 745646618 | 74478156419 | 745155031 | 99.93% | 692839898 | 92.98% | 18678956 | 2.51% | 23.71 |
| PU8268 | 637814965 | 63739198931 | 623445898 | 97.75% | 586403163 | 94.06% | 66200478 | 10.62% | 18.23 |
| PU8284 | 465716674 | 46540881220 | 465397041 | 99.93% | 439859436 | 94.51% | 20212379 | 4.34% | 14.57 |
| PU8308 | 456718639 | 45631701898 | 456536858 | 99.96% | 437568639 | 95.85% | 29103590 | 6.37% | 13.98 |
| PU8314 | 531811987 | 53150094598 | 531340142 | 99.91% | 501780395 | 94.44% | 33136054 | 6.24% | 16.31 |
| PU8321 | 325161300 | 32499724451 | 324795932 | 99.89% | 308142314 | 94.87% | 257191262 | 79.19% | 2.20 |
| PU8328 | 519795054 | 51947294011 | 519455692 | 99.93% | 490680036 | 94.46% | 22275505 | 4.29% | 16.27 |
| PU8336 | 483230108 | 48291749130 | 482800398 | 99.91% | 454733673 | 94.19% | 37369041 | 7.74% | 14.57 |
| PU8354 | 508851552 | 50861096258 | 508503209 | 99.93% | 487817882 | 95.93% | 109056144 | 21.45% | 13.08 |
| PU8389 | 232958221 | 23278308014 | 232504644 | 99.81% | 218148110 | 93.83% | 40896758 | 17.59% | 6.25 |
| PU8449 | 706433094 | 70593557892 | 705644832 | 99.89% | 670824412 | 95.07% | 103106039 | 14.61% | 19.70 |
| PU8450 | 544789585 | 54427690669 | 544475015 | 99.94% | 510275186 | 93.72% | 115313711 | 21.18% | 14.02 |
| PU8460 | 485476621 | 48517984883 | 485182094 | 99.94% | 460186128 | 94.85% | 25423988 | 5.24% | 15.05 |
| PU8473 | 303290728 | 30297783868 | 303164163 | 99.96% | 291496407 | 96.15% | 17506658 | 5.77% | 9.33 |
| PU8488 | 491832556 | 49153636210 | 491472030 | 99.93% | 460712872 | 93.74% | 10151403 | 2.07% | 15.76 |
| PU8494 | 528257756 | 52789930819 | 527944952 | 99.94% | 502391005 | 95.16% | 120477928 | 22.82% | 13.32 |
| PU8500 | 925235392 | 92447157891 | 924676152 | 99.94% | 870349688 | 94.12% | 27619274 | 2.99% | 29.31 |
| PU8501 | 341789212 | 34163912609 | 341549254 | 99.93% | 326634193 | 95.63% | 155017165 | 45.39% | 6.10 |
| PU8527 | 477445126 | 47712430142 | 476925039 | 99.89% | 448864469 | 94.12% | 37846477 | 7.94% | 14.37 |
| PU8532 | 659006677 | 65830153397 | 658612769 | 99.94% | 625460822 | 94.97% | 93714391 | 14.23% | 18.42 |
| PU8559 | 465482242 | 46514680973 | 465148681 | 99.93% | 443282171 | 95.30% | 89132211 | 19.16% | 12.29 |
| PU8562 | 525897722 | 52459023120 | 525580029 | 99.94% | 495400455 | 94.26% | 87287935 | 16.61% | 14.21 |
| PU8569 | 564211015 | 56394183566 | 563788430 | 99.93% | 533749210 | 94.67% | 161387942 | 28.63% | 13.18 |
| PU8578 | 548187227 | 54791896952 | 547909142 | 99.95% | 519866716 | 94.88% | 228509664 | 41.71% | 10.45 |
| PU8580 | 674642001 | 67408693325 | 674298907 | 99.95% | 630936071 | 93.57% | 17555835 | 2.60% | 21.48 |
| PU8587 | 488227539 | 48791647362 | 487943353 | 99.94% | 460723804 | 94.42% | 33117702 | 6.79% | 14.88 |
| PU8590 | 595867900 | 59538925812 | 595303845 | 99.91% | 559481729 | 93.98% | 32451911 | 5.45% | 18.40 |
| PU8595 | 443144585 | 44283649187 | 442818706 | 99.93% | 418715705 | 94.56% | 16461566 | 3.72% | 13.95 |
| PU8603 | 600805943 | 60052385870 | 600387770 | 99.93% | 571529040 | 95.19% | 163353417 | 27.21% | 14.31 |
| PU8613 | 368975355 | 36874653320 | 368570233 | 99.89% | 348203191 | 94.47% | 30970202 | 8.40% | 11.04 |
| PU8614 | 221685094 | 22156455132 | 221161584 | 99.76% | 208603967 | 94.32% | 57205458 | 25.87% | 5.36 |
| PU8615 | 532402272 | 53205404262 | 531975059 | 99.92% | 501861708 | 94.34% | 31119047 | 5.85% | 16.39 |
| PU8616 | 546824658 | 54654031612 | 546387699 | 99.92% | 518730228 | 94.94% | 355211755 | 65.01% | 6.24 |
| PU8618 | 318802027 | 31860105579 | 318510383 | 99.91% | 300954359 | 94.49% | 16975078 | 5.33% | 9.87 |
| PU8619 | 714530640 | 71419773422 | 714078531 | 99.94% | 682962049 | 95.64% | 353687913 | 49.53% | 11.79 |
| PU8620 | 643992969 | 64329386521 | 643592322 | 99.94% | 617370699 | 95.93% | 55272924 | 8.59% | 19.22 |
| PU8628 | 520652383 | 52008967615 | 520406183 | 99.95% | 495513946 | 95.22% | 110061470 | 21.15% | 13.37 |
| PU8635 | 515195689 | 51478612334 | 514933849 | 99.95% | 483970361 | 93.99% | 10982743 | 2.13% | 16.48 |
| PU8641 | 439743898 | 43947195350 | 439473665 | 99.94% | 413623580 | 94.12% | 16667896 | 3.79% | 13.83 |
| PU8647 | 653201943 | 65270934308 | 652911291 | 99.96% | 622714439 | 95.38% | 90108853 | 13.80% | 18.40 |
| PU8653 | 563933444 | 56349946259 | 563557797 | 99.93% | 529713929 | 93.99% | 20143676 | 3.57% | 17.77 |
| PU8656 | 528983022 | 52861147282 | 528686321 | 99.94% | 498508551 | 94.29% | 24671540 | 4.67% | 16.49 |
| PU8659 | 385141116 | 38490445614 | 384852278 | 99.93% | 361051857 | 93.82% | 154441454 | 40.13% | 7.54 |
| PU8664 | 712951710 | 71250504287 | 712579839 | 99.95% | 672042022 | 94.31% | 43747161 | 6.14% | 21.89 |
| PU8671 | 470943477 | 47063271893 | 470703178 | 99.95% | 443480779 | 94.22% | 18161102 | 3.86% | 14.80 |
| PU8679 | 494940363 | 49448236189 | 494551698 | 99.92% | 465989587 | 94.22% | 122023251 | 24.67% | 12.13 |
| PU8681 | 600657168 | 60028292422 | 600344711 | 99.95% | 568780139 | 94.74% | 29547164 | 4.92% | 18.68 |
| PU8682 | 469579976 | 46930735850 | 469166882 | 99.91% | 442011774 | 94.21% | 17698074 | 3.77% | 14.77 |
| PU8683 | 640936137 | 64061771788 | 640629559 | 99.95% | 606461143 | 94.67% | 84138150 | 13.13% | 18.20 |
| PU8686 | 475713307 | 47549497281 | 475344078 | 99.92% | 452185527 | 95.13% | 133614188 | 28.11% | 11.18 |
| PU8690 | 648653578 | 64213204588 | 647893029 | 99.88% | 595798469 | 91.96% | 64019748 | 9.88% | 18.39 |
| PU8698 | 656871947 | 65658463267 | 656426290 | 99.93% | 630550225 | 96.06% | 358471914 | 54.61% | 9.74 |
| PU8699 | 528870931 | 52858341563 | 528541601 | 99.94% | 500302856 | 94.66% | 222489127 | 42.09% | 10.01 |
| PU8703 | 694260934 | 69391666611 | 693658738 | 99.91% | 663628893 | 95.67% | 536868236 | 77.40% | 5.11 |
| PU8712 | 518751940 | 51848838748 | 518452348 | 99.94% | 497403397 | 95.94% | 153348135 | 29.58% | 11.95 |
| PU8717 | 525705173 | 52538675277 | 521628183 | 99.22% | 493002593 | 94.51% | 13708468 | 2.63% | 16.63 |
| PU8719 | 388859730 | 38863049027 | 380881429 | 97.95% | 359098697 | 94.28% | 17152858 | 4.50% | 11.91 |
| PU8721 | 786612050 | 78608664739 | 785985765 | 99.92% | 751657934 | 95.63% | 161878654 | 20.60% | 20.36 |
| PU8731 | 699628380 | 69350182504 | 699193996 | 99.94% | 651944929 | 93.24% | 28912016 | 4.14% | 21.32 |
| PU8732 | 689651649 | 68917388101 | 689227246 | 99.94% | 650635619 | 94.40% | 33309440 | 4.83% | 21.46 |
| PU8742 | 609912951 | 60960705557 | 609585565 | 99.95% | 583814555 | 95.77% | 214998669 | 35.27% | 12.92 |
| PU8745 | 541112853 | 54086490684 | 540769466 | 99.94% | 512468397 | 94.77% | 133182927 | 24.63% | 13.34 |
| PU8747 | 380571896 | 38028574156 | 380277819 | 99.92% | 361340542 | 95.02% | 72779676 | 19.14% | 10.05 |
| PU8748 | 529944117 | 52963173778 | 529568101 | 99.93% | 505736803 | 95.50% | 113493109 | 21.43% | 13.61 |
| PU8752 | 408344523 | 40751772267 | 408120990 | 99.95% | 387691046 | 94.99% | 106927002 | 26.20% | 9.81 |
| PU8768 | 1443913160 | 144299066291 | 1442844561 | 99.93% | 1361691757 | 94.38% | 93137736 | 6.46% | 44.15 |
| PU8771 | 827642668 | 82703406765 | 826705341 | 99.89% | 775203945 | 93.77% | 45049308 | 5.45% | 25.55 |
| PU8772 | 298966082 | 29876303313 | 298799375 | 99.94% | 282498985 | 94.54% | 73491845 | 24.60% | 7.36 |
| PU8775 | 350766754 | 35055603712 | 350566542 | 99.94% | 331986497 | 94.70% | 15332076 | 4.37% | 10.98 |
| PU8780 | 354904802 | 35472302909 | 348109878 | 98.09% | 329997814 | 94.80% | 22738081 | 6.53% | 10.65 |
| PU8783 | 586963807 | 58642432419 | 586520789 | 99.92% | 554533850 | 94.55% | 257821248 | 43.96% | 10.73 |
| PU8789 | 204265682 | 20416540905 | 204121050 | 99.93% | 193341873 | 94.72% | 7878566 | 3.86% | 6.43 |
| PU8792 | 827279041 | 82654209097 | 826631515 | 99.92% | 774222758 | 93.66% | 29948383 | 3.62% | 26.02 |
| PU8794 | 354529460 | 35432400629 | 354119337 | 99.88% | 333696013 | 94.23% | 23828165 | 6.73% | 10.80 |
| PU8797 | 632315971 | 63202559522 | 631811956 | 99.92% | 604443651 | 95.67% | 503184452 | 79.64% | 4.19 |
| PU8799 | 807766591 | 80694965736 | 807281589 | 99.94% | 774751189 | 95.97% | 109823122 | 13.60% | 22.78 |
| PU8811 | 466019559 | 46576204256 | 465784308 | 99.95% | 439379603 | 94.33% | 21207330 | 4.55% | 14.56 |
| PU8815 | 585419966 | 58496150344 | 585083296 | 99.94% | 555721615 | 94.98% | 82759065 | 14.14% | 16.41 |
| PU8817 | 370549615 | 37031890068 | 370285884 | 99.93% | 350405189 | 94.63% | 20773582 | 5.61% | 11.43 |
| PU8818 | 458713505 | 45840902118 | 458012708 | 99.85% | 431515962 | 94.21% | 59891024 | 13.08% | 13.02 |
| PU8819 | 688503820 | 68805584950 | 687750976 | 99.89% | 647964447 | 94.21% | 43485427 | 6.32% | 21.08 |
| PU8821 | 256435368 | 25629445430 | 256232554 | 99.92% | 242427365 | 94.61% | 10946071 | 4.27% | 8.03 |
| PU8822 | 359505476 | 35926271296 | 359201208 | 99.92% | 338386858 | 94.21% | 15943884 | 4.44% | 11.23 |
| PU8823 | 477275840 | 47698543412 | 476932704 | 99.93% | 450749406 | 94.51% | 24180244 | 5.07% | 14.82 |
| PU8824 | 692156561 | 69169826945 | 691576003 | 99.92% | 654448689 | 94.63% | 61834911 | 8.94% | 20.60 |
| PU8825 | 545769787 | 54542812328 | 545476070 | 99.95% | 514894671 | 94.39% | 28005744 | 5.13% | 16.94 |
| PU8826 | 660075072 | 65973480163 | 659578632 | 99.92% | 620961319 | 94.15% | 437988967 | 66.40% | 7.24 |
| PU8833 | 600419920 | 60009055783 | 600007879 | 99.93% | 565013948 | 94.17% | 153843169 | 25.64% | 14.59 |
| PU8834 | 510630380 | 51032723823 | 497991354 | 97.52% | 470115520 | 94.40% | 46817856 | 9.40% | 14.77 |
| PU8838 | 800832406 | 80003633251 | 800419125 | 99.95% | 751023701 | 93.83% | 43843453 | 5.48% | 24.71 |
| PU8842 | 638211271 | 63769680907 | 637693230 | 99.92% | 600030429 | 94.09% | 19751744 | 3.10% | 20.20 |
| PU8845 | 476869963 | 47651991833 | 476561320 | 99.94% | 449405509 | 94.30% | 29750585 | 6.24% | 14.61 |
| PU8846 | 144606722 | 14376114241 | 144516139 | 99.94% | 137673963 | 95.27% | 11625717 | 8.04% | 4.26 |
| PU8851 | 534373307 | 53399547085 | 533882694 | 99.91% | 503017568 | 94.22% | 27965913 | 5.24% | 16.55 |
| PU8855 | 587802428 | 58746879289 | 587496387 | 99.95% | 562630618 | 95.77% | 111809832 | 19.03% | 15.56 |
| PU8856 | 262060835 | 26115074466 | 261922166 | 99.95% | 249151575 | 95.12% | 32867115 | 12.55% | 7.36 |
| PU8863 | 457512089 | 45687613065 | 457223389 | 99.94% | 428708977 | 93.76% | 100590560 | 22.00% | 11.64 |
| PU8864 | 538471772 | 53810191039 | 537793676 | 99.87% | 510585254 | 94.94% | 339697598 | 63.17% | 6.45 |
| PU8865 | 259819756 | 25968543009 | 259263501 | 99.79% | 244147506 | 94.17% | 79890361 | 30.81% | 5.86 |
| PU8867 | 371820041 | 37150819983 | 371414456 | 99.89% | 350523321 | 94.38% | 133708301 | 36.00% | 7.77 |
| PU8869 | 703429534 | 70308368392 | 702925391 | 99.93% | 668995805 | 95.17% | 172175903 | 24.49% | 17.35 |
| PU8876 | 452456837 | 45212960022 | 452095492 | 99.92% | 425395967 | 94.09% | 19453314 | 4.30% | 14.15 |
| PU8878 | 590501601 | 59024197043 | 590192927 | 99.95% | 562351264 | 95.28% | 203417735 | 34.47% | 12.66 |
| PU8882 | 480266472 | 48006981659 | 479839094 | 99.91% | 460982925 | 96.07% | 383995541 | 80.03% | 3.12 |
| PU8885 | 631752810 | 63137127801 | 631312271 | 99.93% | 599246242 | 94.92% | 28974876 | 4.59% | 19.72 |
| PU8887 | 623394344 | 62290582576 | 622953282 | 99.93% | 583345452 | 93.64% | 72227127 | 11.59% | 18.01 |
| PU8904 | 1017123409 | 101649156879 | 1016634804 | 99.95% | 970064208 | 95.42% | 224469648 | 22.08% | 25.91 |
| PU8916 | 473983073 | 47368349301 | 473764727 | 99.95% | 447421418 | 94.44% | 18412283 | 3.89% | 14.91 |
| PU8918 | 393714907 | 39346410284 | 393342214 | 99.91% | 370985002 | 94.32% | 21149665 | 5.38% | 12.18 |
| PU8919 | 465858252 | 46552925014 | 464516779 | 99.71% | 438904013 | 94.49% | 40563607 | 8.73% | 13.87 |
| PU8922 | 649166521 | 64877083903 | 642139800 | 98.92% | 606310261 | 94.42% | 44252797 | 6.89% | 19.56 |
| PU8924 | 611349904 | 61104104302 | 610942686 | 99.93% | 574015349 | 93.96% | 215039039 | 35.20% | 12.96 |
| PU8925 | 357420409 | 35722266522 | 357245104 | 99.95% | 336967270 | 94.32% | 72699678 | 20.35% | 9.31 |
| PU8926 | 636089478 | 63575647814 | 635620158 | 99.93% | 598272796 | 94.12% | 215220178 | 33.86% | 13.76 |
| PU8929 | 631865961 | 63154976754 | 631337097 | 99.92% | 600247755 | 95.08% | 448631559 | 71.06% | 5.96 |
| PU8938 | 582325043 | 57476537485 | 582123991 | 99.97% | 541955521 | 93.10% | 46609258 | 8.01% | 16.77 |
| PU8944 | 499563949 | 49931927853 | 499253201 | 99.94% | 471824089 | 94.51% | 263535635 | 52.79% | 7.71 |
| PU8948 | 536441122 | 53611407497 | 515105191 | 96.02% | 485415997 | 94.24% | 87949782 | 17.07% | 13.98 |
| PU8950 | 218465219 | 21823994603 | 218132506 | 99.85% | 205736769 | 94.32% | 40500601 | 18.57% | 5.79 |
| PU8954 | 419705248 | 41941058744 | 419494473 | 99.95% | 395475818 | 94.27% | 78319714 | 18.67% | 11.16 |
| PU8956 | 539646154 | 53941550240 | 539331438 | 99.94% | 508800828 | 94.34% | 175571599 | 32.55% | 11.91 |
| PU8959 | 635045441 | 63468776840 | 634619850 | 99.93% | 598578174 | 94.32% | 27452264 | 4.33% | 19.87 |
| PU8960 | 360317359 | 36015444672 | 360076331 | 99.93% | 340667045 | 94.61% | 18975923 | 5.27% | 11.18 |
| PU8964 | 939346644 | 93676801330 | 938741952 | 99.94% | 887226346 | 94.51% | 86971780 | 9.26% | 27.63 |
| PU8965 | 638258992 | 63782419427 | 637866148 | 99.94% | 599825893 | 94.04% | 346966675 | 54.39% | 9.49 |
| PU8969 | 507719506 | 50743432052 | 507493941 | 99.96% | 478261853 | 94.24% | 80322554 | 15.83% | 13.98 |
| PU8970 | 695288519 | 69487475343 | 694819884 | 99.93% | 658579598 | 94.78% | 67731793 | 9.75% | 20.52 |
| PU8977 | 238521240 | 23836160867 | 238371666 | 99.94% | 224250580 | 94.08% | 88718925 | 37.22% | 4.89 |
| **mean** | 518346722 | 51773617406 | 517000611 | 99.71% | 488532496 | 94.50% | 100529872 | 19.39% | 13.6 |

# Table S3

**Baseline characteristics of laboratory test results between noncritical and critical patients.** Laboratory results were also obtained from the initial laboratory test during admission. After excluding the laboratory parameters with a missing ratio greater than 30% across 399 patients in the entire cohort, results of 62 laboratory tests were compiled in the current analysis. Values were presented as mean ± standard deviation (SD) and the two-side Mann–Whitney U test was used to conduct comparison.

| **Laboratory parameter** | **Total(n=399)** | **Noncritical (n=345)** | **Critical (n=54)** | **P value** | **Reference range** | **Unit** | **Abbr** |
| --- | --- | --- | --- | --- | --- | --- | --- |
| Potassium* | 4.15±0.47 | 4.15±0.44 | 4.11±0.62 | 0.1237 | 3.5-5.3 | mmol/L | K |
| Sodium* | 138.98±3.70 | 138.75±2.45 | 140.40±7.79 | 0.1252 | 137-147 | mmol/L | Na |
| Chloride* | 100.93±4.11 | 100.94±3.47 | 100.87±6.95 | 0.2689 | 96-108 | mmol/L | Cl |
| Anion gap# | 9.82±2.80 | 9.81±2.47 | 9.94±4.4 | 0.3920 | 8-16 | mmol/L | AG |
| Magnesium§ | 0.86±0.08 | 0.86±0.08 | 0.83±0.09 | 0.0018 | 0.65-1.25 | mmol/L | Mg |
| Phosphate§ | 1.09±0.26 | 1.11±0.23 | 0.95±0.37 | <0.0001 | 0.9-1.34 | mmol/L | P |
| Calcium§ | 2.11±0.13 | 2.13±0.12 | 2.03±0.18 | <0.0001 | 2.0-2.5 | mmol/L | Ca |
| AST/ALT | 0.89±0.43 | 0.88±0.42 | 0.95±0.51 | 0.1433 | NA | NA | AST/ALT |
| Aspartate aminotransferase | 29.26±45.36 | 25.57±15.67 | 52.78±114.92 | 0.0141 | 8-40 | U/L | AST |
| Alanine transaminase | 43.59±81.92 | 38.05±36.5 | 78.94±200.68 | 0.1873 | 5-40 | U/L | ALT |
| Gamma Glutamyl Transpeptidase | 44.33±45.45 | 42.78±44.97 | 54.24±47.6 | 0.0391 | 11-60 | U/L | γ-GT |
| Total bilirubin | 11.84±18.18 | 10.32±5.26 | 21.58±46.81 | 0.0002 | 3-20 | μmol/L | TBil |
| Direct bilirubin | 4.03±11.69 | 3.02±2.57 | 10.48±30.56 | <0.0001 | 1.7-6.8 | μmol/L | DBil |
| Indirect bilirubin | 7.82±6.94 | 7.30±3.32 | 11.14±16.65 | 0.0359 | 3.4-12.0 | μmol/L | I-Bil |
| Total protein | 66.15±6.53 | 66.82±5.99 | 61.89±8.09 | <0.0001 | 60-83 | g/L | TP |
| Albumin | 38.63±5.27 | 39.32±4.66 | 34.21±6.68 | <0.0001 | 33-55 | g/L | Alb |
| Globulin | 27.52±4.84 | 27.50±4.58 | 27.68±6.27 | 0.3074 | 20-35 | g/L | Glb |
| Albumin/Globulin | 1.45±0.35 | 1.47±0.32 | 1.32±0.50 | <0.0001 | 1.5-2.5 | NA | A/G |
| Prealbumin | 301.28±90.46 | 313.60±81.97 | 222.53±102.6 | <0.0001 | 150-400 | mg/L | PA |
| Total bile acid | 5.55±4.66 | 5.27±4.23 | 7.33±6.59 | 0.0243 | 0-10 | μmol/L | TBA |
| Blood urea nitrogen§ | 5.71±3.23 | 5.39±2.63 | 7.79±5.37 | 0.0001 | 2.9-8.2 | mmol/L | BUN |
| Creatinine§ | 70.53±65.18 | 69.84±55.02 | 74.98±111.29 | 0.0040 | 57-111 | μmol/L | Gre |
| Uric acid§ | 276.47±100.02 | 289.12±96.20 | 194.18±84.81 | <0.0001 | 208-428 | μmol/L | UA |
| Cystatin C§ | 0.99±0.53 | 0.96±0.50 | 1.14±0.69 | 0.0070 | 0.55-1.05 | mg/L | CysC |
| White blood cell count† | 6.89±4.80 | 6.65±4.64 | 8.47±5.54 | 0.0080 | 3.5-9.5 | ×10^9/L | WBC |
| Red blood cell count† | 3.85±0.67 | 3.95±0.61 | 3.23±0.73 | <0.0001 | 3.8-5.1 | ×10^12/L | RBC |
| Hemoglobin† | 118.21±20.66 | 121.31±18.65 | 97.94±21.85 | <0.0001 | 130-175 | g/L | Hb |
| Hematocrit† | 35.93±5.82 | 36.80±5.27 | 30.26±6.05 | <0.0001 | 35-45 | % | HCT |
| Mean corpuscular volume† | 93.64±5.51 | 93.53±5.20 | 94.37±7.26 | 0.0167 | 82-100 | fl | MCV |
| Mean corpuscular hemoglobin† | 30.74±1.97 | 30.79±1.88 | 30.40±2.48 | 0.2433 | 27-34 | pg | MCH |
| Mean corpuscular hemoglobin concentration† | 328.23±9.36 | 329.14±8.50 | 322.31±12.25 | <0.0001 | 316-354 | g/L | MCHC |
| Platelet count§§ | 220.15±79.91 | 225.55±75.32 | 184.94±98.89 | 0.0020 | 125-350 | G/L | PLT |
| Neutrophil percentage† | 63.44±12.83 | 61.77±11.33 | 74.30±16.41 | <0.0001 | 40-75 | % | NP |
| Lymphocyte percentage† | 25.89±10.90 | 27.20±9.80 | 17.35±13.61 | <0.0001 | 20-50 | % | LP |
| Monocytes percentage† | 7.20±2.69 | 7.42±2.46 | 5.76±3.58 | <0.0001 | 3-10 | % | MP |
| Eosinophil percentage† | 3.00±2.97 | 3.13±2.95 | 2.17±3.01 | <0.0001 | 0.4-8.0 | % | EP |
| Basophil percentage† | 0.47±0.37 | 0.47±0.37 | 0.41±0.37 | 0.0380 | 0-1 | % | BP |
| Neutrophil count† | 4.53±3.14 | 4.18±2.41 | 6.84±5.54 | <0.0001 | 1.8-6.3 | ×10^9/L | NC |
| Lymphocyte count† | 1.70±3.08 | 1.80±3.28 | 1.05±0.66 | <0.0001 | 1.1-3.2 | ×10^9/L | LC |
| Monocytes count† | 0.45±0.20 | 0.46±0.18 | 0.41±0.26 | 0.066 | 0.1-0.6 | ×10^9/L | MC |
| Eosinophil count† | 0.19±0.22 | 0.19±0.22 | 0.14±0.2 | 0.0003 | 0.20-0.52 | ×10^9/L | EC |
| Basophil count† | 0.03±0.02 | 0.03±0.02 | 0.03±0.02 | 0.3714 | 0.00-0.06 | ×10^9/L | BC |
| Red blood cell volume distribution width-CV† | 13.84±1.56 | 13.68±1.45 | 14.87±1.88 | <0.0001 | 12.1-14.3 | % | RDW-CV |
| Platelet distribution width† | 16.11±0.43 | 16.08±0.40 | 16.36±0.51 | <0.0001 | 9.9-15.4 | % | PDW |
| Mean platelet volume† | 9.70±1.179 | 9.59±1.06 | 10.40±1.6 | 0.0001 | 9.1-11.9 | fl | MPV |
| Plateletcrit† | 0.21±0.07 | 0.21±0.06 | 0.18±0.09 | 0.0197 | 0.2-0.4 | % | PCT |
| Total cholesterol¶ | 4.75±1.31 | 4.80±1.29 | 4.29±1.35 | 0.0006 | 0-5.2 | mmol/L | TC |
| Triglyceride¶ | 1.79±1.60 | 1.82±1.66 | 1.50±0.54 | 0.0006 | 0-1.7 | mmol/L | TG |
| High density lipoprotein cholesterol¶ | 1.15±0.34 | 1.16±0.34 | 1.00±0.34 | 0.0051 | 1.04-1.66 | mmol/L | HDL |
| Low density lipoprotein cholesterol¶ | 2.78±0.97 | 2.80±0.96 | 2.54±1.03 | 0.0001 | 0-3.12 | mmol/L | LDL |
| Apolipoprotein A-I†† | 1.08±0.29 | 1.09±0.28 | 0.95±0.31 | 0.0047 | 1-1.6 | g/L | apoAI |
| Apolipoprotein B†† | 1.01±0.28 | 1.01±0.27 | 0.98±0.3 | <0.0001 | 0.6-1.2 | g/L | apoB |
| Lipoprotein (a)†† | 27.28±29.39 | 27.66±29.75 | 23.24±25.35 | 0.0002 | 0-30 | mg/dL | Lp(a) |
| Alkaline phosphatase | 67.13±21.69 | 65.19±19.42 | 79.56±30.07 | 0.0001 | 40-150 | U/L | AKP |
| Lactate dehydrogenase | 204.20±127.60 | 186.29±60.61 | 318.63±288.07 | <0.0001 | 109-245 | U/L | LDH |
| Creatine kinase‡ | 62.85±46.65 | 64.21±47.09 | 48.40±39.59 | 0.0416 | 24-194 | U/L | CK |
| Creatine kinase-MB‡ | 11.80±7.29 | 11.81±7.43 | 11.73±5.77 | <0.0001 | 0-25 | U/L | CK-MB |
| α-Hydroxybutyrate dehydrogenase‡ | 156.05±57.23 | 151.90±55.08 | 200.03±61.95 | <0.0001 | 72-182 | U/L | α-HBDH |
| Blood glucose§ | 6.27±2.14 | 6.09±1.94 | 7.42±2.93 | 0.0003 | 3.9-6.1 | mmol/L | BG |
| Total carbon dioxide§ | 28.26±3.46 | 28.03±2.94 | 29.82±5.59 | 0.0035 | 20-29 | mmol/L | TCO2 |
| C-reactive protein\|\| | 12.88±29.95 | 7.45±16.96 | 47.65±58.78 | <0.0001 | 0.0-8.0 | mg/L | CRP |
| Osmolality# | 298.20±9.06 | 297.28±5.35 | 304.10±19.79 | 0.0123 | 280-310 | mOsm/L | OSM |
| Abbr: Abbreviation, NA: not available.  *Missing in 4 noncritical patients.  #Missing in 4 noncritical patients and 1 critical patient.  §Missing in 1 critical patient.  †Missing in 5 noncritical patients and 2 critical patients.  §§Missing in 6 noncritical patients and 2 critical patients.  ¶ Missing in 35 noncritical patients and 25 critical patients.  ††Missing in 36 noncritical patients and 25 critical patients.  ‡ Missing in 27 noncritical patients and 24 critical patients.  \|\|Missing in 12 noncritical patients and 2 critical patients. | | | | | | | |

# Table S4

**Comparison of performance towards critical COVID-19 prediction among different models.** All evaluation metrics were calculated based on the independent testing dataset in the 100 iterations of random training/testing splits. CI: confidence interval.

|  | Sensitivity% (95% CI) | Specificity% (95% CI) | PPV%  (95% CI) | NPV%  (95% CI) | MCC%  (95% CI) |
| --- | --- | --- | --- | --- | --- |
| LABModel | 68.5  (45.5-90.9) | 87.8  (79.7-95.7) | 46.8  (33.3-71.0) | 94.7  (90.8-98.4) | 48.4  (27.3-73.4) |
| FRAGLModel | 57.4  (31.6-81.8) | 73.6  (58.7-82.6) | 25.4  (15.6-34.9) | 91.7  (87.0-96.2) | 23.0  (3.3-40.4) |
| TSSModel | 83.3  (58.9-100.0) | 90.1  (82.6-94.2) | 57.0  (39.4-72.4) | 97.2  (93.2-100.0) | 63.1  (41.3-81.6) |
| MOTIFModel | 68.5  (36.4-90.9) | 83.5  (73.9-88.4) | 39.4  (25.0-50.0) | 94.4  (89.5-98.2) | 41.9  (18.4-58.9) |
| M2Model | 85.2  (63.6-100.0) | 93.3  (86.2-98.6) | 66.7  (48.8-88.9) | 97.6  (94.0-100.0) | 71.0  (49.8-88.8) |

# Table S5

**Gene set enrichment analysis of the** **TSS-associated genes showing many of them were linked to COVID-19.** These TSS-associated genes were identified by M2Model. Only significant genes were shown (P<0.05).

| **COVID-19 related gene set description** | **Genes** | **Overlap** | **P value** | **GSE** |
| --- | --- | --- | --- | --- |
| SARS-CoV perturbation; 7 Up Genes from GEN3VA; Human Vero E6 cells | TNFAIP3 | 1/7 | 0.006 | GSE30589 |
| 500 genes up-regulated by SARS-CoV-2 in human cardiomyocytes | NCF1;LSMEM1;TNFAIP3 | 3/461 | 0.008 | GSE150392 |
| 500 genes up-regulated by SARS-CoV-2 in human hiPSC-CMs cells at 24h | NCF1;LSMEM1;TNFAIP3 | 3/490 | 0.009 | GSE150392 |
| SARS-CoV perturbation; 23 Up Genes from GEN3VA; Human Vero E6 cells | TNFAIP3 | 1/23 | 0.021 | GSE30589 |
| Coronavirus Perturbation; 326 Up Genes from GEN3VA Human Calu-3 2B4 cells | ZNF484;TNFAIP3 | 2/266 | 0.023 | GSE45042 |
| SARS perturbation; 388 Up Genes from GEN3VA; Human airway epithelium (HAE) cells | NCF1;TNFAIP3 | 2/342 | 0.037 | GSE47961 |
| Top 500 up genes for SARS-CoV-2 infection in Mesocricetus auratus hamster blood Day 2 | GSDMD;TNFAIP3 | 2/363 | 0.042 | GSE162208 |
| Top 500 up genes for SARS-CoV-2 infection in Mesocricetus auratus hamster lung Day 3 | GSDMD;TNFAIP3 | 2/370 | 0.043 | GSE162208 |
| Top 500 up genes for SARS-CoV-2 infection in Mesocricetus auratus hamster lung Day 5 | GSDMD;TNFAIP3 | 2/377 | 0.044 | GSE162208 |
| Top 500 upregulated genes in human nasal epithelial cells with SARS-CoV-2 infection (Mutant, 72 hpi) | TNFAIP3;PPP2R2A | 2/398 | 0.049 | GSE162131 |
| Top 500 upregulated genes in mouse kidney with SARS-CoV-2 infection (Day 3) | GSDMD;TNFAIP3 | 2/399 | 0.049 | GSE162113 |

# Table S6

**Optimal hyperparameter values determined by Bayesian optimization method for LABModel, FRAGLModel, TSSModel, MOTIFModel, and M2Model, respectively.** Parameter descriptions for LightGBM may be found on LightGBM website. Default values were used for parameters otherwise.

|  | Parameter | Search space† | Optimal value | | | | |
| --- | --- | --- | --- | --- | --- | --- | --- |
|  |  |  | LABModel | FRAGLModel | TSSModel | MOTIFModel | M2Model |
| LightGBM | num_iterations | Discrete values in [80, 300], step=20 | 260 | 240 | 240 | 140 | 200 |
|  | num_leaves | Discrete values in [10, 300], step=2. | 154 | 162 | 218 | 130 | 102 |
|  | max_depth | Discrete values in [3, 10], step=1 | 3 | 8 | 6 | 6 | 5 |
|  | min_data_in_leaf | Discrete values in [2, 60], step=1 | 21 | 54 | 29 | 8 | 45 |
|  | learning_rate | Discrete values in [0.01, 0.5], step=0.01 | 0.29 | 0.22 | 0.16 | 0.14 | 0.4 |
|  | bagging_fraction | Discrete values in [0.6, 1], step=0.1 | 0.7 | 0.8 | 0.8 | 0.6 | 0.9 |
|  | feature_fraction | Discrete values in [0.3, 0.8], step=0.1 | 0.7 | 0.6 | 0.4 | 0.3 | 0.6 |
|  | reg_alpha | Continuous values in [0.01, 0.1] | 0.0928 | 0.0494 | 0.0641 | 0.0953 | 0.0686 |
|  | reg_lamda | Continuous values in [0.01, 0.1] | 0.0589 | 0.082 | 0.0632 | 0.0989 | 0.0232 |
| Focal loss | alpha | Continuous values in [0.1, 0.75] | 0.3507 | 0.3295 | 0.2841 | 0.2866 | 0.152 |
|  | gamma | Continuous values in [0.5, 5] | 0.6161 | 3.4263 | 0.9096 | 1.4037 | 0.7038 |

† Parameter values in search space were chosen uniformly.

# Table S7

**Microsoft Excel spreadsheet file with raw data from all 399 patients with COVID-19 in this study.**

(**A**) Raw data of clinical laboratory results (LAB features) from all 399 patients with COVID-19 in the study.

(**B**) Raw data of cfDNA fragment length ratio per $\sim$10.4 bp bin (FRAGL features) for all 399 patients with COVID-19 in the study.

(**C**) Raw data of transcription start site coverage score (TSS features) for all 399 patients with COVID-19 in the study. Columns represented TSS positions that can be mapped to the associated gene symbols using (E).

(**D**) Raw data of frequency of 4-nucleotide (4-mer) motifs at 5’ fragment ends (MOTIF features) for all 399 patients with COVID-19 in the study.

(**E**) Transcription start site (TSS) positions and gene symbols mapping. The TSS positions were downloaded from <http://hgdownload.cse.ucsc.edu/goldenpath/hg38/database/refGene.txt.gz>.

**Supplementary figure legends**


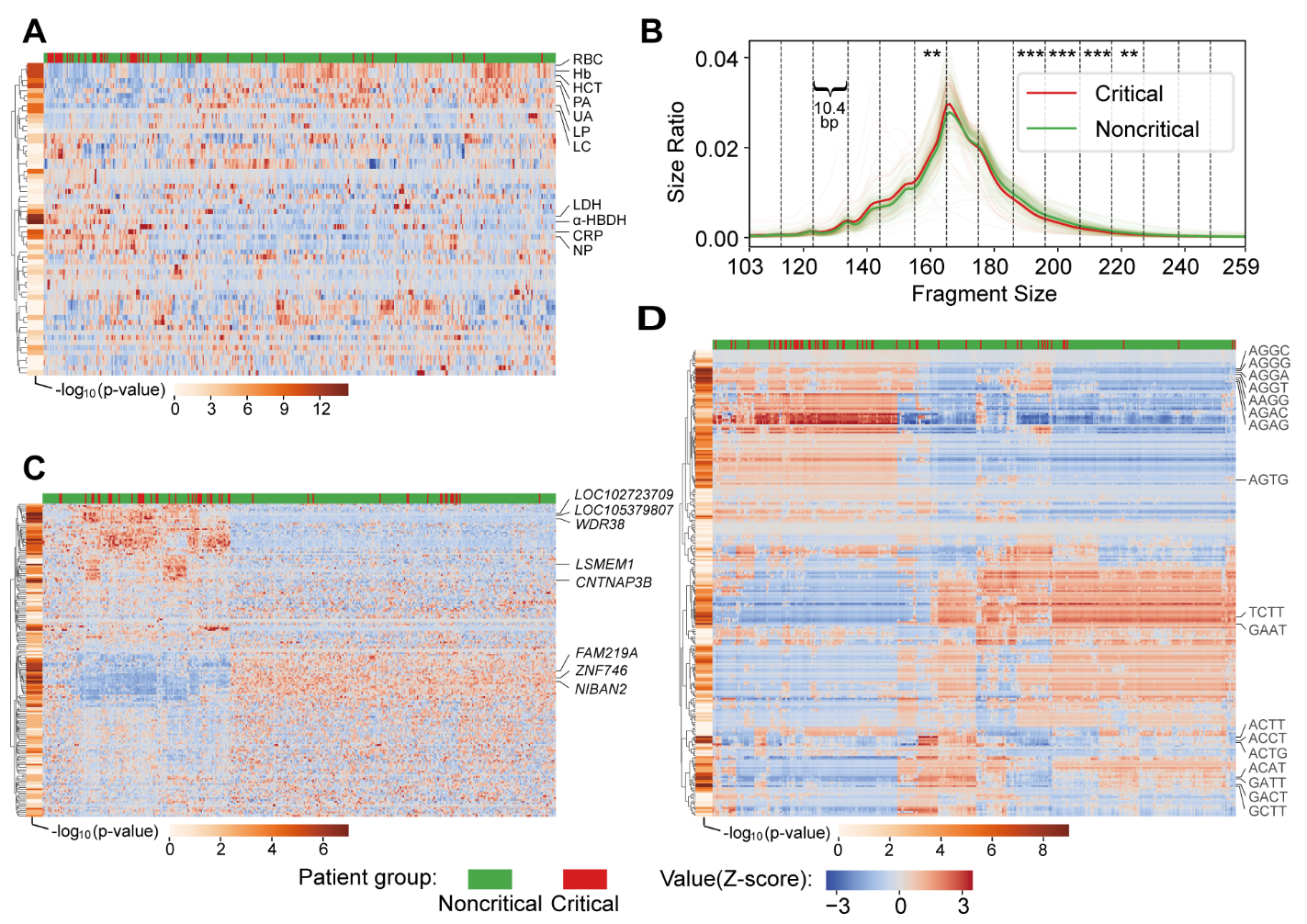


# Figure S1

**Analyzing distributions of each type of features after data preprocessing between the two patient groups.** (**A**, **C, and D**) Hierarchical clustering analysis of (A) 62 LAB features, (C) 177 TSS features and (D) 256 MOTIF features showing distinct separation between critical and noncritical patients. TSS features were represented by the associated gene symbols. All values were z-scored. Significantly different LAB features [$-\log_{10}(P-value)$>8], TSS features [$-\log_{10}(P-value)$>6] and MOTIF features [$-\log_{10}(P-value)$>7] were highlighted, respectively. Statistical test: two-side Mann-Whitney U test. RBC: red blood cell count, Hb: hemoglobin, HCT: hematocrit, PA: prealbumin, UA: uric acid, LP: lymphocyte percentage; LC: lymphocyte count, LDH: lactate dehydrogenase, α-HBDH: α-hydroxybutyrate dehydrogenase, CRP: C-reactive protein, NP: neutrophil percentage. (**B**) Fragment size profiles of cfDNA from critical and noncritical patients . The red line represented the average fragment length in cfDNA of critical patients while the green line represented that of noncritical ones. A peak in average fragment length for both critical and noncritical patients was nearly 166 bp. A total of 15 FRAGL features were derived from each of nearly 10.4 bp bins. Statistical test: two-side Mann-Whitney U test. ***P*<0.01, ****P*<0.001. The raw values of the four types of features can be found in **Table S7A-D**.

**
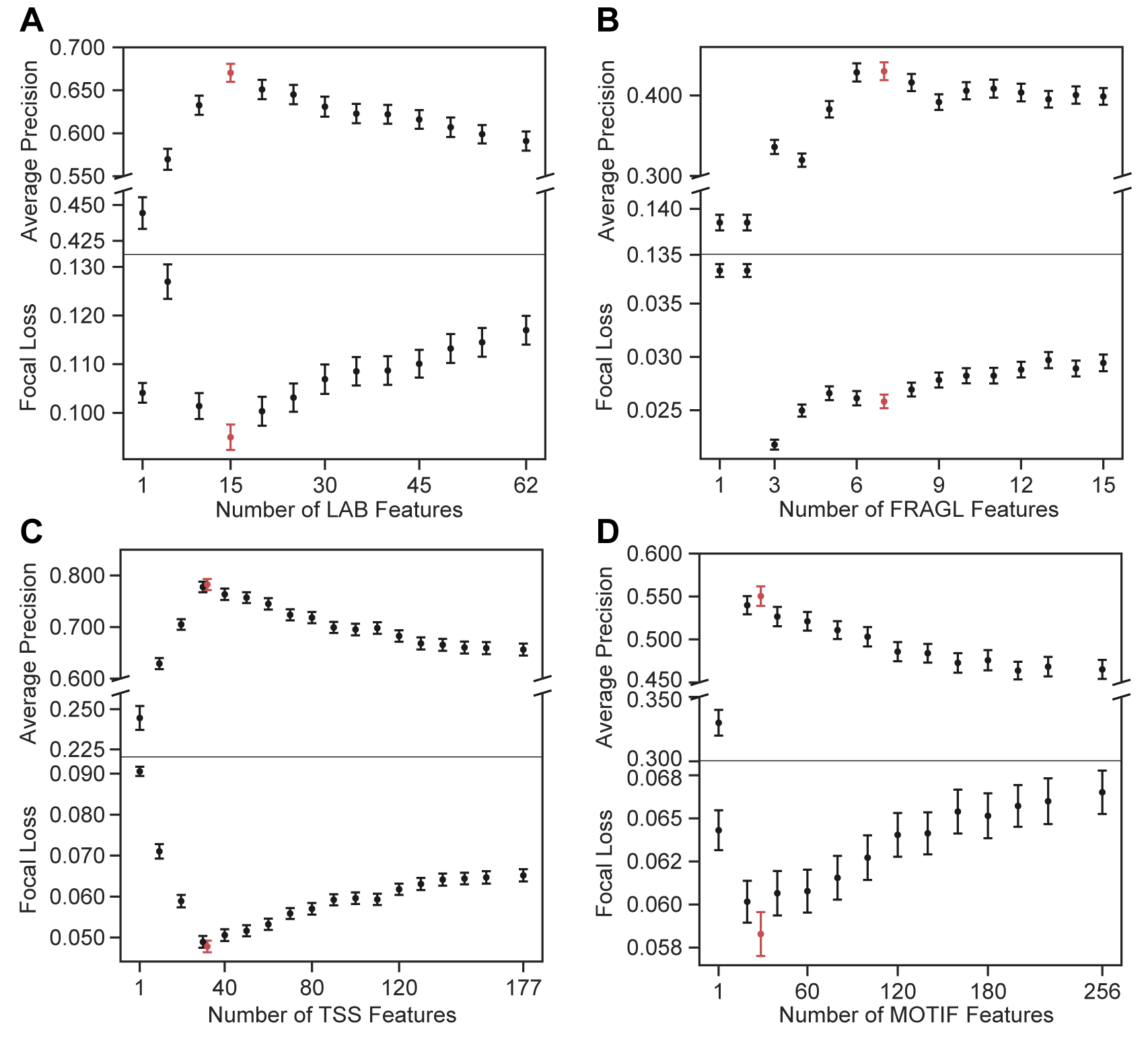
**

# Figure S2

**Prediction performance in terms of average precision score and focal loss with respect to the number of ranked single-type features.** Error bar in red represented the optimal top features determined by (**A**) LABModel, (**B**) FRAGLModel, (**C**) TSSModel, and (**D**) MOTIFModel. Error bars: mean ± standard error (SE).


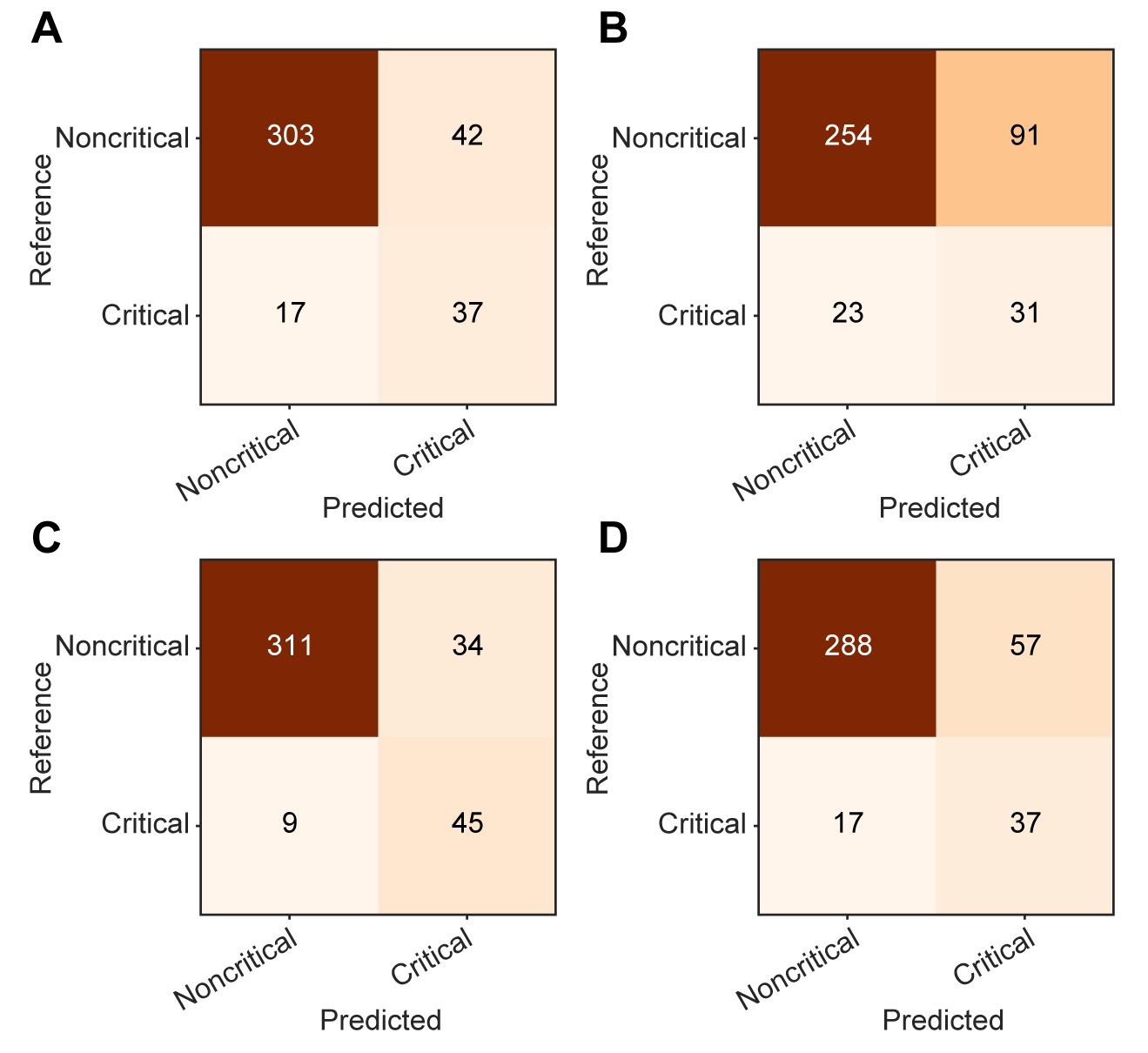


# Figure S3

**Confusion matrices of single-type feature-based classifier showing performance in distinguishing critical from noncritical COVID-19 patients.** Each of them was calculated as the mean of predicted probabilities across 100 iterations against the corresponding optimal cutoff as shown in (Figure 1D). (**A**) Confusion matrix resulting from LABModel. (**B**) Confusion matrix resulting from FRAGLModel. (**C**) Confusion matrix resulting from TSSModel. (**D**) Confusion matrix resulting from MOTIFModel.

**
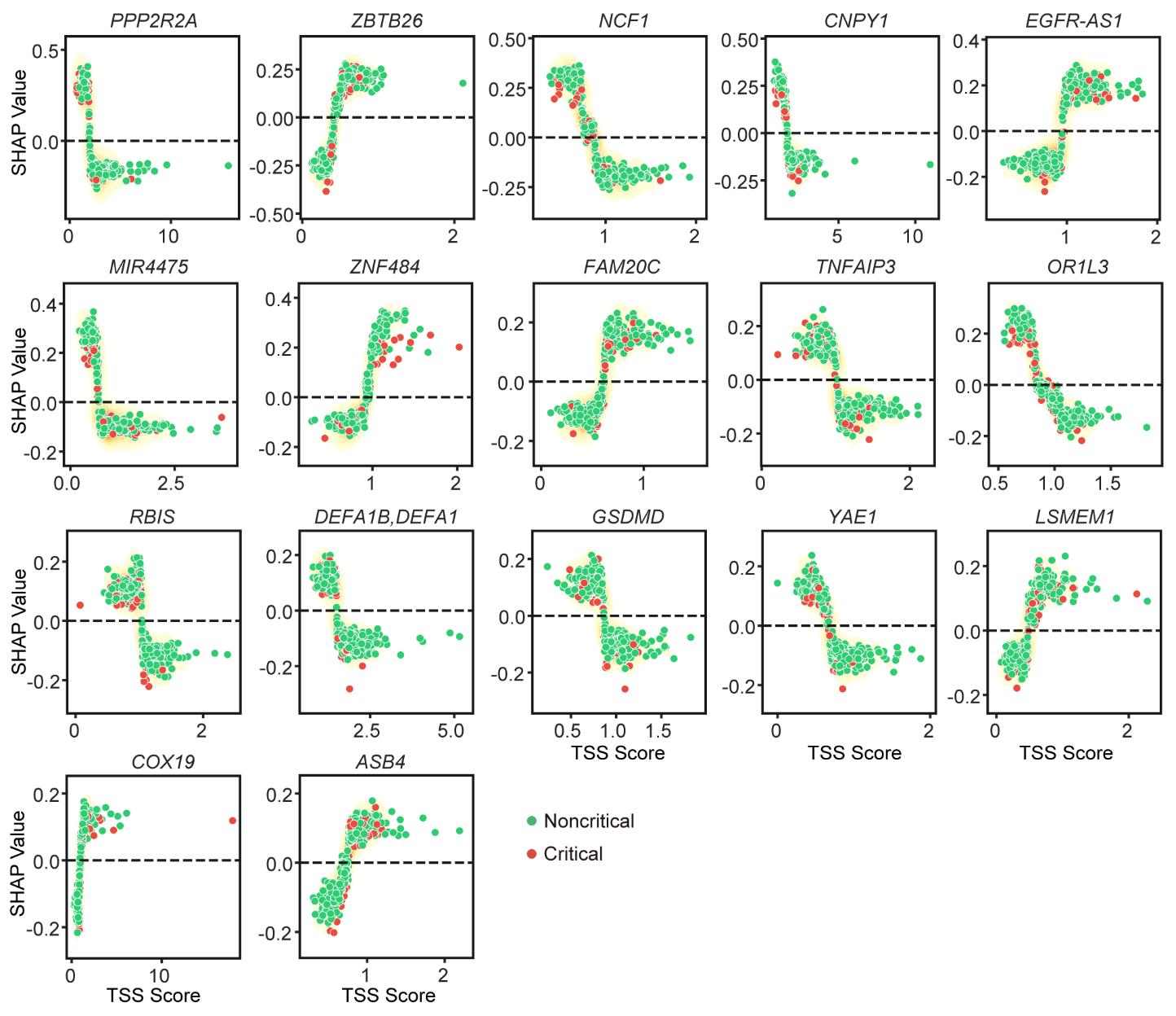
**

# Figure S4

**Nonlinear relationships between the top 17 TSS features identified by M2Model and the contributions towards critical COVID-19 prediction.**


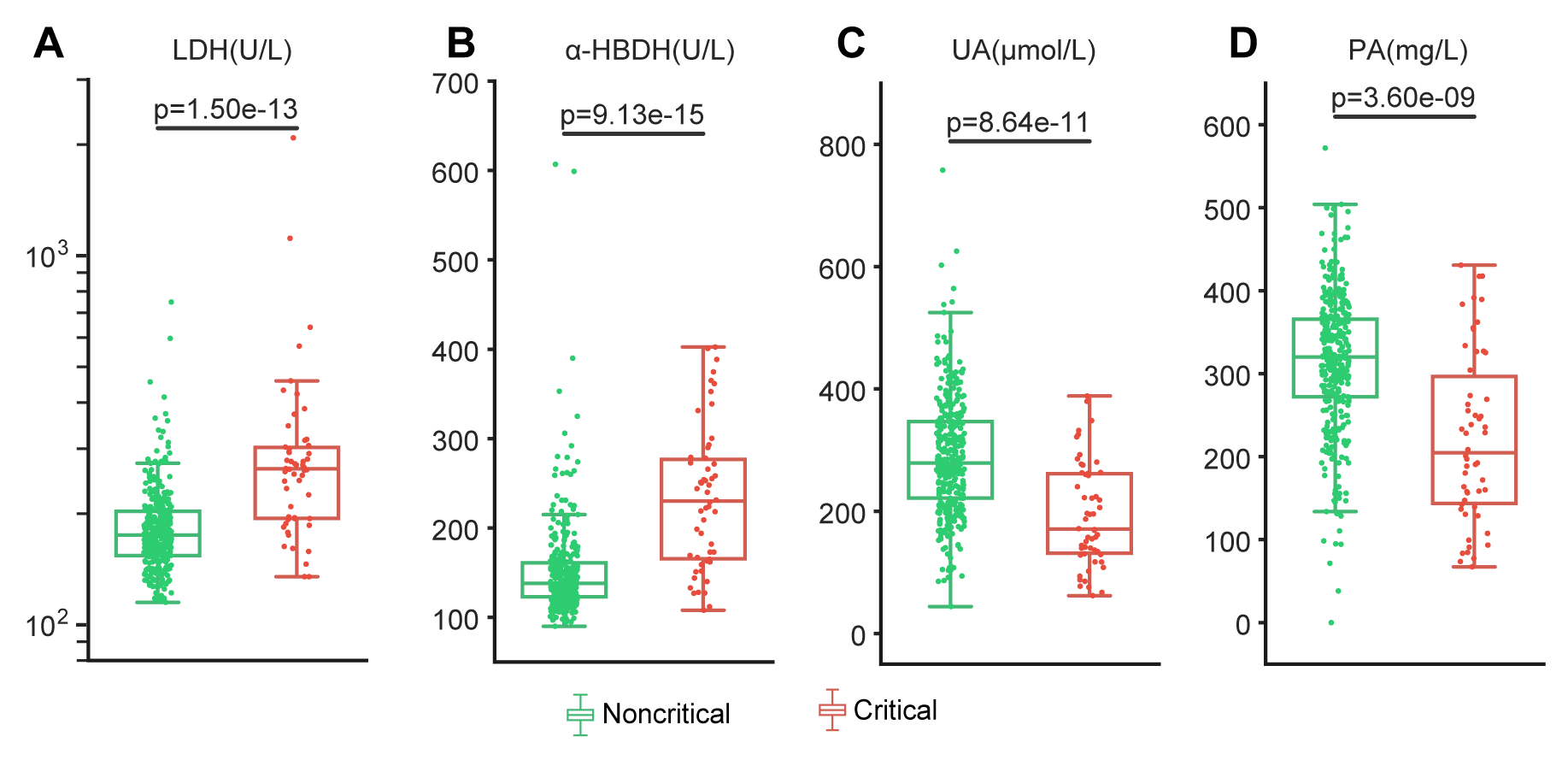


# Figure S5

**Distributions of the top 4 LAB features identified by M2Model between critically ill patients and noncritically ill patients.** (A) Distribution of lactate dehydrogenase (LDH). (B) Distribution of α-hydroxybutyrate dehydrogenase (α-HBDH). (C) Distribution of uric acid (UA). (D) Distribution of prealbumin (PA). Boxplots: each box corresponded to an interval from the 25th to 75th percentile (interquartile range, IQR) and the median, whiskers = 1.5 $\boldsymbol{\times}$ IQR. Statistical test: two-side Mann-Whitney U test.


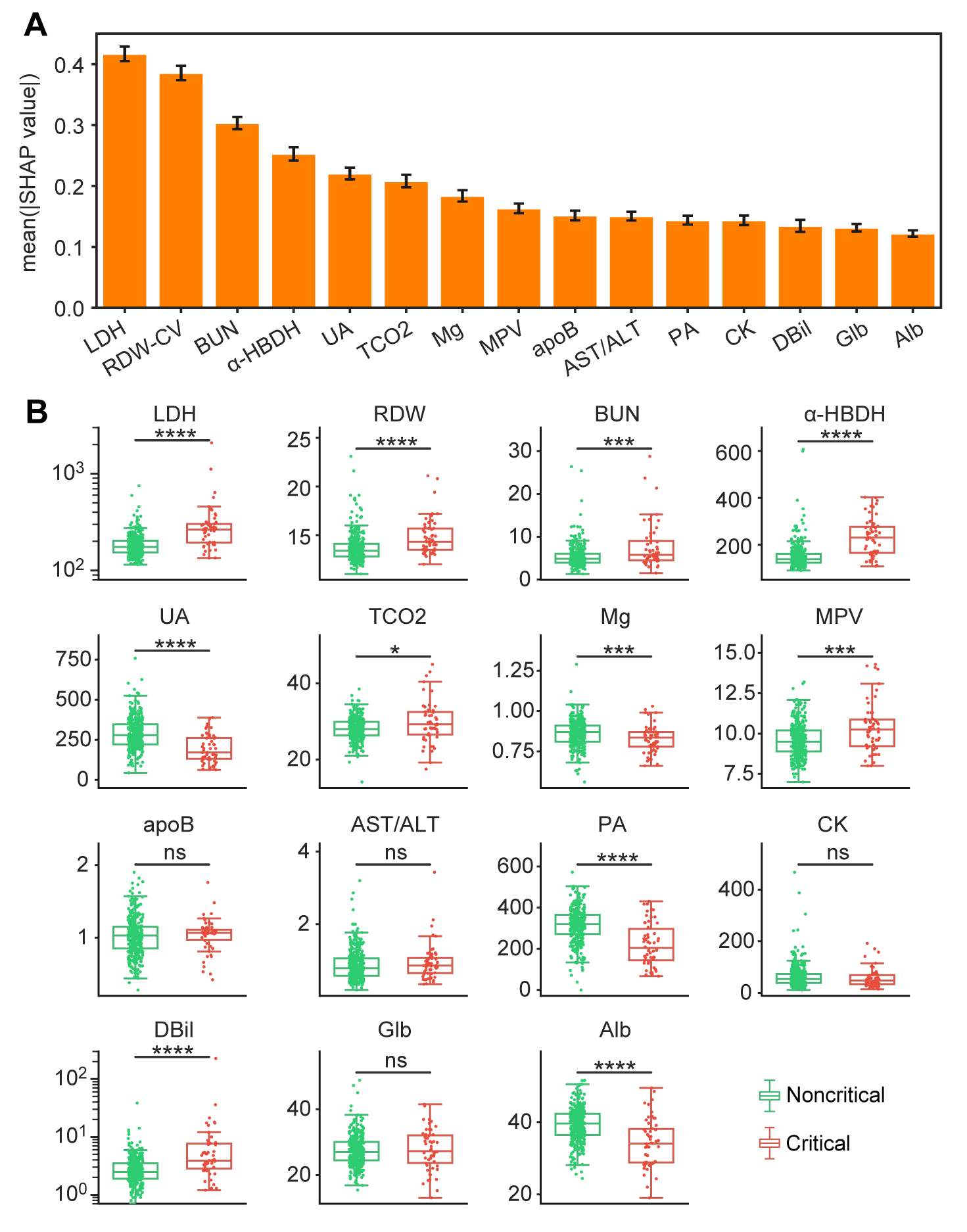


# Figure S6

**Top 15 LAB features prioritized by LABModel and ranked by the mean absolute SHAP values.** (**A**) Top LAB features ranked by the mean absolute SHAP values. Error bars: mean ± standard error (SE). (**B**) Distribution of top LAB features between critically ill patients and noncritically ill patients. LDH: lactate dehydrogenase (U/L), RDW-CV: red blood cell volume distribution width-CV (%), BUN: blood urea nitrogen (mmol/L), α-HBDH: α-hydroxybutyrate dehydrogenase (U/L), UA: uric acid (μmol/L), TCO2: total carbon dioxide (mmol/L), Mg: magnesium (mmol/L), MPV: mean platelet volume (fl), apoB: apolipoprotein B (g/L), AST/ALT: aspartate aminotransferase/alanine transaminase, PA: prealbumin (mg/L), CK: creatine kinase (U/L), DBil: direct bilirubin (μmol/L), Glb: globulin (g/L), Alb: albumin (g/L). Boxplots: each box corresponded to an interval from the 25th to 75th percentile (interquartile range, IQR) and the median, whiskers = 1.5 $\boldsymbol{\times}$ IQR. Statistical test: two-side Mann-Whitney U test. ns: not significant, **P*<0.05, ***P*<0.01, ****P*<0.001, *****P*<0.0001.


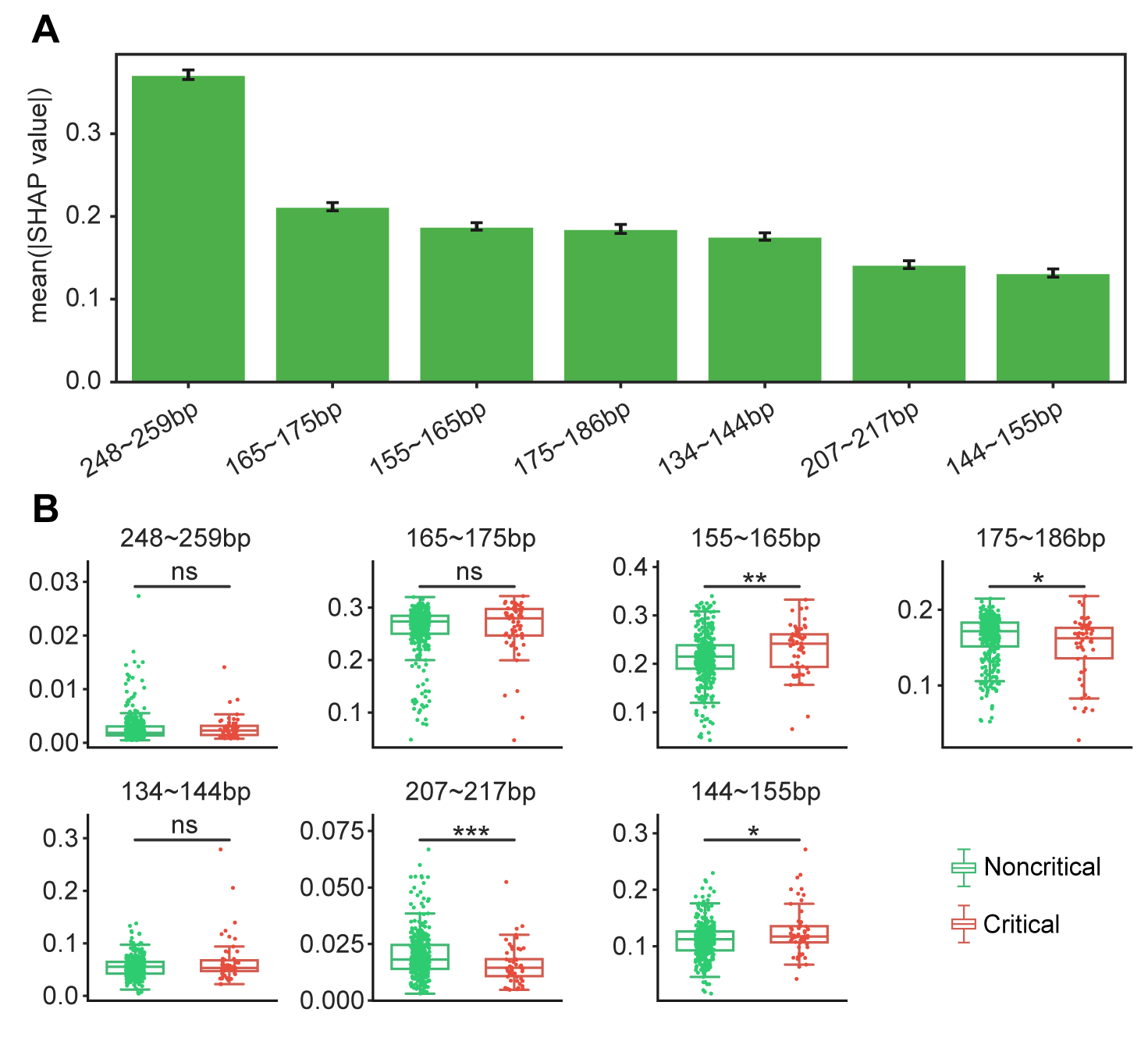


# Figure S7

**Top 7 FRAGL features prioritized by FRAGLModel and ranked by the mean absolute SHAP values.** (**A**) Top FRAGL features ranked by the mean absolute SHAP values. Error bars: mean ± standard error (SE). (**B**) Distribution of top FRAGL features between critically ill patients and noncritically ill patients. Boxplots: each box corresponded to an interval from the 25th to 75th percentile (interquartile range, IQR) and the median, whiskers = 1.5 $\boldsymbol{\times}$ IQR. Statistical test: two-side Mann-Whitney U test. ns: not significant, **P*<0.05, ***P*<0.01, ****P*<0.001.


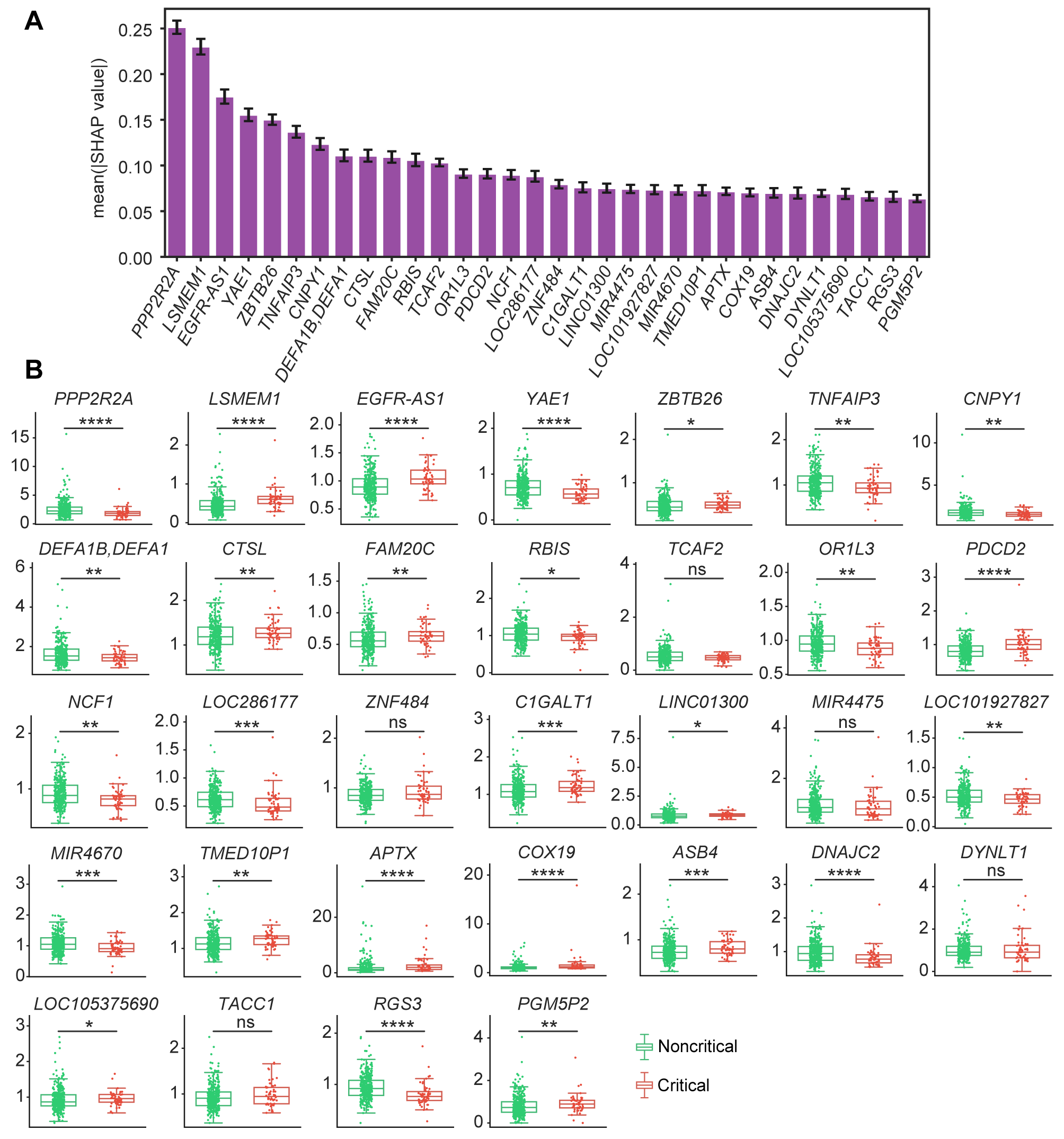


# Figure S8

**Top 32 TSS features prioritized by TSSModel and ranked by the mean absolute SHAP values.** (**A**) Top TSS features ranked by the mean absolute SHAP values. Error bars: mean ± standard error (SE). TSS features were represented by the associated gene symbols. (**B**) Distribution of top TSS features between critically ill patients and noncritically ill patients. Boxplots: each box corresponded to an interval from the 25th to 75th percentile (interquartile range, IQR) and the median, whiskers = 1.5 $\boldsymbol{\times}$ IQR. Statistical test: two-side Mann-Whitney U test. ns: not significant, **P*<0.05, ***P*<0.01, ****P*<0.001, *****P*<0.0001.


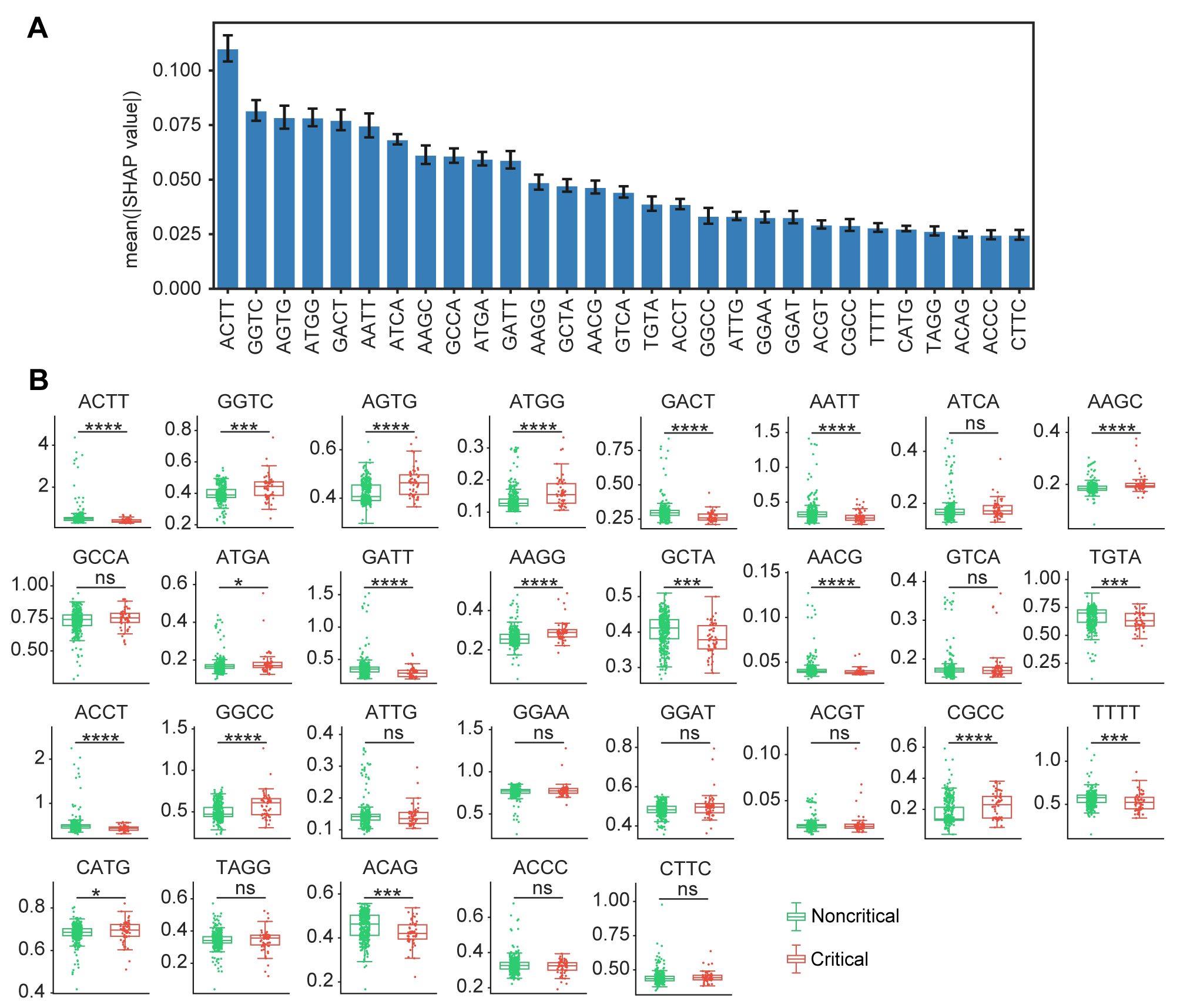


# Figure S9

**Top 29 MOTIF features prioritized by MOTIFModel and ranked by the mean absolute SHAP values.** (**A**) Top MOTIF features ranked by the mean absolute SHAP values. Error bars: mean ± standard error (SE). (**B**) Distribution of top MOTIF features between critically ill patients and noncritically ill patients. Boxplots: each box corresponded to an interval from the 25th to 75th percentile (interquartile range, IQR) and the median, whiskers = 1.5 $\boldsymbol{\times}$ IQR. Statistical test: two-side Mann-Whitney U test. ns: not significant, **P*<0.05, ****P*<0.001, *****P*<0.0001.


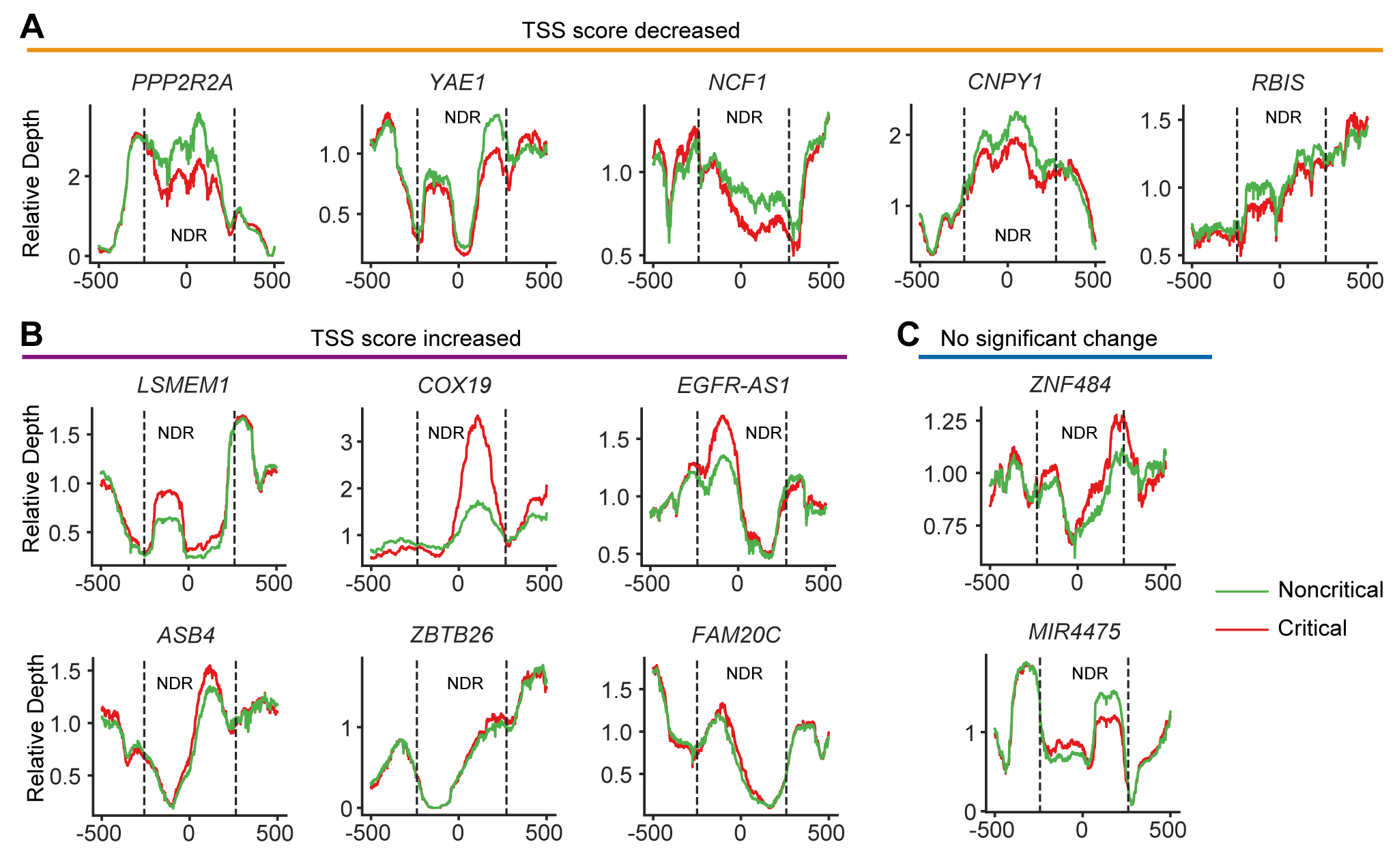


# Figure S10

**The difference in coverage depth of sequencing reads related to TSSs identified by M2Model between critically and noncritically ill patients.** Relative coverage depths in the range from -500 bp to +500 bp centered on the TSSs with (**A**) decreased TSS coverage scores, (**B**) increased TSS coverage scores, and (**C**) no significant change in TSS coverage scores were shown. Red line and green line represented the average coverage depth across critical patients and noncritical patients, respectively. Nucleosome-depleted region (NDR, -250 bp to +250 bp of TSS) was displayed between the dashed lines.


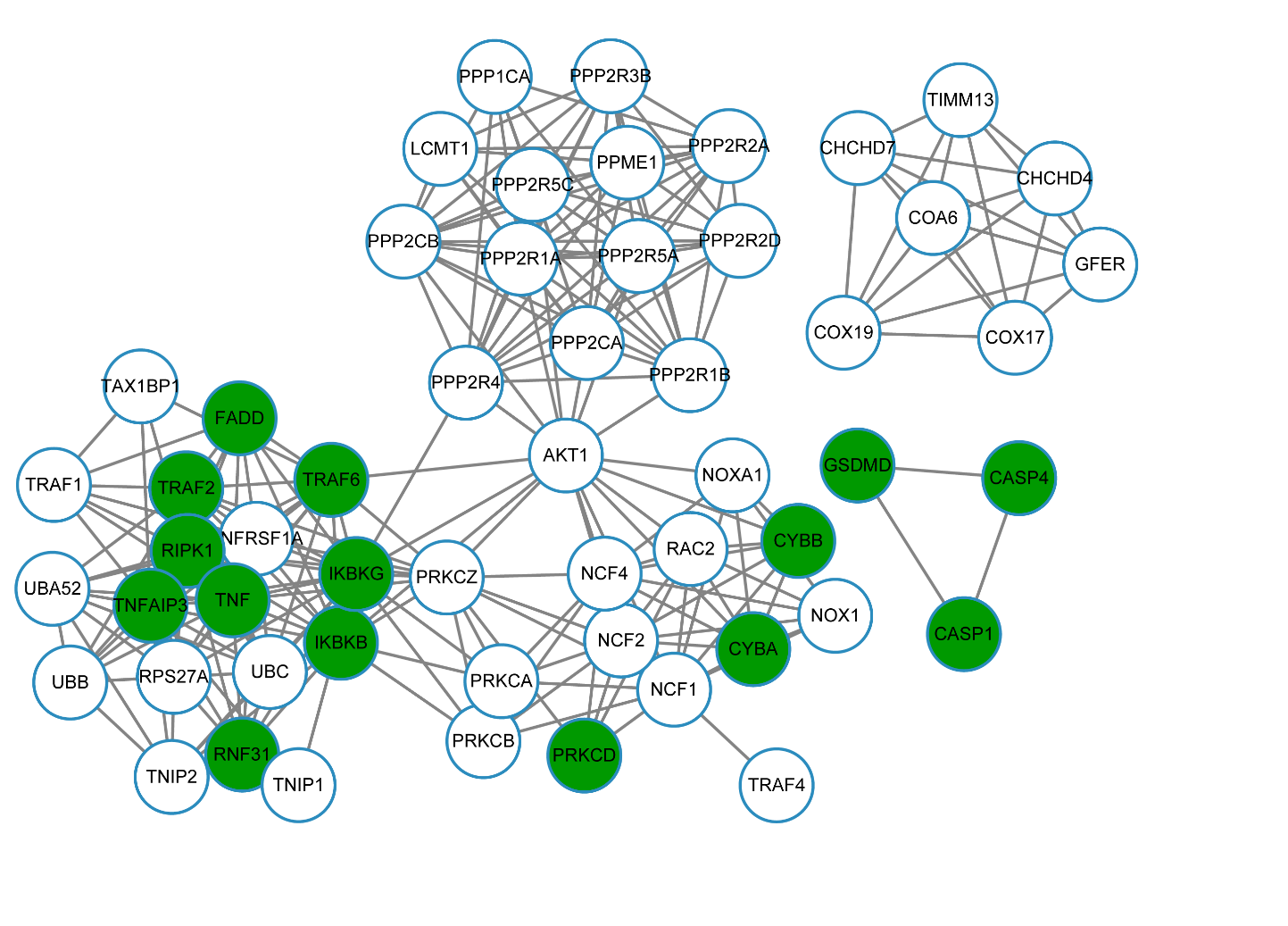


# Figure S11

**STRING protein-protein interaction (PPI) network of prioritized proteins.** Green nodes indicated molecules in the most significant “NOD-like receptor signaling pathway”. STRING: Search Tool for the Retrieval of Interacting Genes/Proteins.
